# Glucocorticoid signalling dysregulation and immune cell adaptation in Anorexia Nervosa

**DOI:** 10.1038/s41380-026-03756-4

**Published:** 2026-07-20

**Authors:** Francesca Mottarlini, Lorenzo Da Dalt, Susanna Parolaro, Andrea Baragetti, Beatrice Rizzi, Sofia Taddini, Anna Chiara Cigognini, Francesca Vairano, Sara Bertelli, Armando D’Agostino, Fabrizia Bonacina, Giuseppe Danilo Norata, Fabio Fumagalli, Lucia Caffino

**Affiliations:** 1 Department of Pharmacological and Biomolecular Sciences “Rodolfo Paoletti”, Università degli Studi di Milano, Via Balzaretti 9, 20133 Milan, Italy; 2 Center for Neuroscience, University of Camerino, Camerino, Italy; 3 Department of Mental Health and Addiction, Asst Santi Paolo e Carlo, Milan, Italy; 4 Department of Health Sciences, Università degli Studi di Milano, Milan, Italy

**Keywords:** Psychiatric disorders, Neuroscience, Biomarkers, Molecular biology

## Abstract

Anorexia Nervosa (AN) is a neuropsychiatric disorder marked by compulsive weight-loss and hyperactivity, with poorly understood underlying mechanisms and limited treatment outcomes. Here we show that women with AN, at the first medical evaluation, exhibit hyperactivity and hypercortisolemia, together with a reduced immune cell count yet paradoxically showing increased levels of cell activation. One year later, only subjects considered in remission showed greater increases in cortisol and cytokine levels, along with enhanced monocyte differentiation and recruitment. Using the activity-based anorexia (ABA) rat model, we reproduced AN core features, including hypercorticosteronemia, and observed innate-skewed immune profiles, as well as persistent microglial and glucocorticoid receptor (GR) dysfunction in the ventral hippocampus. Pharmacological blockade of GR with RU486 attenuated hyperactivity and reshaped microglial phenotype in the ventral hippocampus. Our results suggest that cortisol elevation and immune cell adaptation may perpetuate disease vulnerability beyond weight normalization, challenging the notion of weight regain as an indicator of remission.

## Introduction

Anorexia Nervosa (AN) is a severe neuropsychiatric disorder that typically emerges in adolescent females [1, 2]. Individuals with AN experience profound disturbance in body image and derive excessive reward from self-imposed starvation and compulsive exercise aimed at achieving weight loss [3]. Many patients develop a chronic and relapsing course of illness [4], which often leads to a life-threatening spiral.

Individuals with AN can endure a chronic state of malnutrition for extended periods while maintaining sufficient energy not only to survive but also to engage in intense physical activity with the aim of losing further weight. Remarkably, these subjects display low incidence of infections [5], a feature that contrasts sharply with other forms of malnutrition [6], suggesting a potential involvement of immune-related dysregulation in AN pathophysiology [7]. Despite its well-defined symptomatology and prolonged course as a major, yet often neglected, clinical and public health concern, the factors that trigger and sustain AN remain a matter of debate. Moreover, the current interventions are hindered by the highly variable clinical presentations and the absence of stage-dependent diagnostic biomarkers.

Our hypothesis builds upon existing evidence suggesting that the life-threatening pursuit of weight loss [8], is driven by miscommunication among multiple biological systems [9]. This underscores a strong interplay between nutritional status and dysregulated peripheral signaling, which culminates in brain dysfunction [10] and may underlie the cognitive disabilities observed in subjects with AN [11, 12], which persist even after weight normalization [13]. In this framework, the Activity-Based Anorexia (ABA) rat model might help in studying AN pathophysiology, as it combines food restriction with free access to a running wheel and recapitulates the core clinical features of the disorder, including voluntary starvation, hyperactivity, and rapid weight loss [14]. These characteristics provide high translational value, making it the gold-standard preclinical tool for investigating the neurobiological mechanisms underlying AN [14, 15]. Interestingly, the combination of severe dieting and hyperactivity triggers a marked dysregulation of both the hypothalamic–pituitary–adrenal (HPA) axis [16–18] and the immune system of peripheral and central origin [19, 20] in both humans and ABA model. Indeed, individuals with AN often exhibit chronic hyperactivation of the HPA axis [17, 21], leading to persistently elevated cortisol levels, the primary stress-related glucocorticoid hormone. We do believe that  this condition, over time, can impair glucocorticoid receptor (GR) sensitivity, reducing the efficacy of cortisol anti-inflammatory actions. As a result, the immune system enters a paradoxical state in which features of immune suppression coexist with low-grade systemic inflammation, as previously observed in chronic stress-related pathological conditions [22, 23].

Glucocorticoids (GCs) are the molecular mediators of the stress response [24], and exert powerful immunomodulatory effects, primarily by suppressing pro-inflammatory cytokine production, inhibiting immune cell proliferation, and maintaining immune homeostasis [24, 25]. As a mechanism, depending on tissue and cell type, GR primarily [26] modulates glucose and lipid metabolism, osteogenesis, immunity, and cognitive processes [26–28] by regulating gene transcription through DNA binding [29]. Maintaining the delicate balance between peripheral GC-mediated signals and proper central GR signal transduction is essential for preserving physiological homeostasis across multiple systems, including both the systemic and central immune response. In this scenario, microglia, i.e., the resident immune cells of the brain, emerge as a critical effector of GCs action [30]. Indeed, GCs modulate microglia activation, proliferation and regulate cytokine production [31]. Through GR signaling, GCs help maintaining microglial homeostasis and prevent excessive neuroinflammation, thereby protecting neural tissue from damage during stress and immune challenges, fundamental to maintain functional neuroplastic mechanisms [32, 33]. Notably, underweight subjects with AN show heightened sensitivity to stress, which is reflected in reduced hippocampal volume [34]. Similarly, in the ABA rat model, significant morphological and molecular changes have been observed in the hippocampus [35], potentially contributing to increased anxiety and cognitive impairments [36]. Indeed, the hippocampus, and specifically its ventral subregion, is crucial for regulating emotions, stress and anxiety responses [37]. Moreover, it is particularly sensitive to GCs during adolescence, a critical period for its maturation and for the emergence of hippocampus-dependent behaviors [38], yet the neuroanatomical substrates underlying these behavioral changes remain poorly understood. We have recently contributed to these findings demonstrating that the induction of the AN phenotype during adolescence, using the ABA paradigm [39, 40], alters the hippocampal non-genomic stress signaling, leading to structural instability and spatial memory deficits in female rats, not restored after weight normalization [41].

Accordingly, and considering the critical gap represented by the lack of stage-dependent biomarkers and clear molecular mechanisms underlying AN pathophysiology, we herein performed a translational investigation by integrating the characterization of the immune cell phenotyping in both humans and rats, with an in depth molecular evaluation of the genomic stress response and microglial transcriptional profile in rats, at different time points of the disease. Our goal was indeed to verify whether hyperactivation of the HPA axis may lead to impaired GR sensitivity, resulting in a dysfunctional GC signaling cascade that fails to properly regulate peripheral immune responses and microglial activity, thereby promoting a persistent neuroimmune imbalance that is maintained despite body weight normalization.

We herein propose a potential mechanism of interaction between the GR system and immune activation through acute pharmacological modulation of GR functionality. Our findings highlight persistent, long-lasting immune- and stress-related signatures of AN that appear to endure even after body weight (BW) normalization. These insights suggest that the biological imprint of AN extends beyond visible recovery, reinforcing the need for treatments targeting its underlying molecular scars.

## Results

### Women with AN are characterized by compulsive exercise

The subjects diagnosed with AN (*n* = 35, Table 1) were evaluated at the first medical evaluation (T0) and after one year (T1). The clinical assessment obtained scoring the Eating Disorder Inventory 3 (EDI-3) subscales (drive for thinness, bulimia, body dissatisfaction) and the Symptom Checklist 90 (SCL90) is reported in Table 1.

**Table 1:** Clinical and anthropometric characteristics of patients with AN and HC; eating disorder psychopathology (EDIs) and the symptomatology (SCL90) evaluations of subjects with AN. For HC, only a single blood draw was performed at baseline (T0).

	HC (*n* = *26*) *Mean* (±SD)	AN T0 (*n* = *35*) *Mean* (±*SD*)	AN T1 (*n* = *20*) *Mean* (±*SD*)	*AN T0 vs AN T1* *p value*
**Age** (*y.o*.)	25,42 (1,90) [23-30]	18,54 (2,20) [16-24]	19,11 (2,25) [17-25]	-
**Age of onset** (*y.o*.)	-	16,84 (1,98)	-	-
**Illness duration** (*m*)	-	20,49 (8,46)	32,28 (8,31)	-
**EDI-3**				
**Drive for thinnes**	-	16,84 (6,24)	15,20 (6,22)	0,5469
**Bulimia**	-	4,81 (2,88)	4,40 (5,32)	0,8223
**Body dissatisfaction**	-	19,85 (7,68)	24,40 (5,68)	0,2295
**SCL90 global score**	-	1,41 (0,64)	1,63 (0,58)	0,6210

Results are presented as raw data ± SD. Unpaired Student’s t test. Statistical significance is calculated at p < 0.05. Detailed statistics are presented in Supplementary Table 3.

**Fig. 1:**
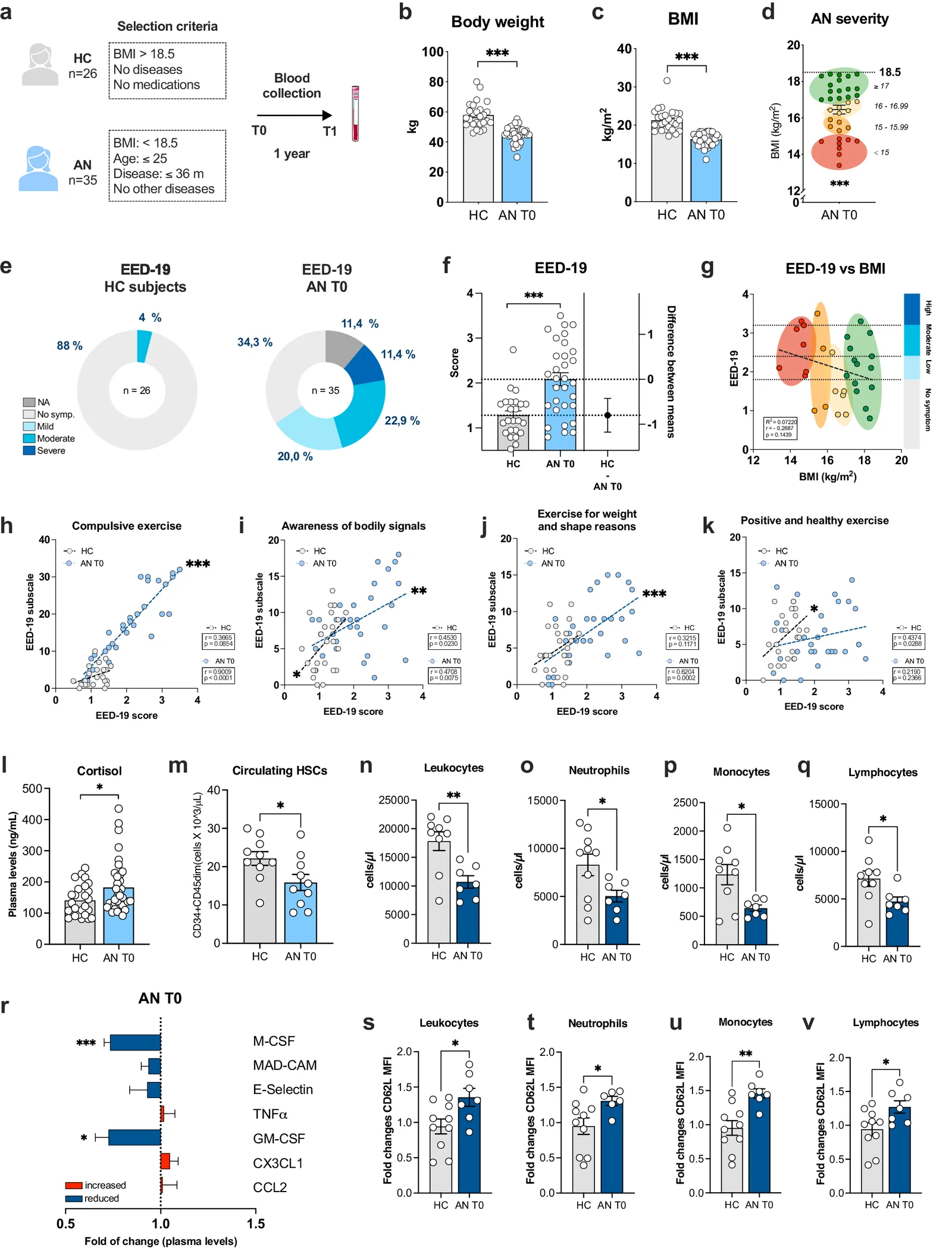
AN subjects with compulsive exercise behaviors show elevated cortisol levels and less, yet activated, immune cells. Experimental timeline **a**. Body weight (BW, **b**) and body mass index (BMI, **c**). Classification of AN severity based on the BMI in four categories (<15 extreme; 15–15,99 severe; 16–16,99 moderate; ≥17,00 mild; **d**). Exercise and eating disorder 19 (EED-19) score distribution in the four categories (severe, moderate, mild and no symptoms) expressed as % in HC (**e**, left) and subjects with AN (**e**, right). Panel **f** shows the EED-19 score of subjects with AN in respect to HC. Pearson’s correlations between the EED-19 and BMI **g**, the categories of compulsive exercise **h**, awareness of body signals **i**, exercise for weight and shape reasons **j**, and positive and healthy exercise **k**, are shown in the same panel for both HC and subjects with AN. Circulating levels of cortisol were measured in plasma and are shown in panel **l**, while hematopoietic stem cells (HSCs, **m**), leukocytes **n**, neutrophils **o**, monocytes **p** and lymphocytes **q** were measured in blood of HC and subjects with AN. CD62L MFI (mean fluorescence intensity) for leukocytes **s**, neutrophils **t**, monocytes **u** and lymphocytes **v** is expressed as fold of changes. Panel **r** shows the levels of cytokines measured in the plasma of HC and subjects with AN and are expressed as fold of changes. Pearson’s product–moment correlation **r** analyses and linear regression analyses (R2) between EED-19 and its relative categories. Unpaired Student’s t test. Statistical significance is calculated at p < 0,05. ^*^p < 0,05, ^**^p < 0,01, ^***^p < 0001. Detailed statistics are presented in Supplementary Table 5.

Compared to HC (*n* = 26) and adjusted for age, subjects with AN at T0 had significantly reduced BW (Fig. 1b) and mean body mass index (BMI), which was below the 18,5 cutoff for underweight [3] (Fig. 1c). We identified 4 subsets of disease severity based on subject’s BMI (Fig. 1d), and 4 clusters based on their Exercise and Eating Disorder 19 (EED-19) score of exercise (Fig. 1e), which was significantly higher than the EED-19 of HC (Fig. 1f), demonstrating that subjects with AN executed higher level of exercise compared to HC. In details, the 54,3% of subjects with AN performed physical activity from mild to severe intensities, while only the 4% of HC presented moderate levels of exercise (Fig. 1f). Among the 54,3% of subjects with AN, 11,4% achieved severe, 22,9% moderate and 20% mild levels of exercise. Moreover, correlating the EED-19 score with patients BMI, we observed a negative trend indicating that patients with lower BMI tended to be higher exercise performers, even though the results were not statistically significant (Fig. 1g). Of note, when analyzing the EED-19 questionnaire subclasses of answers to define the subject’s attitude toward the physical exercise (e.g. negative or positive) and to dissect which of the categories had the major contribution to the global EED-19, we found significant positive correlations in patients with AN between the EED-19 score and the subclasses of answers falling into the compulsive exercise (Fig. 1h), awareness of body signals (Fig. 1i), exercise for weight and shape reasons (Fig. 1j) categories, but not for the positive and healthy exercise (Fig. 1k), supporting their compulsive and unhealthy behavior toward the exercise. Conversely, HC subjects did not show any significant correlation with the only exception of the positive and healthy exercise category (Fig. 1k).

### Patients with AN exhibit hematologic and metabolic abnormalities, hypercortisolism and leukopenia, yet showing signs of activated immune cells

A comparative analysis of hematological parameters (Table 2) showed reduced RBC counts in patients with AN at T0 compared to HC, with partial recovery at T1. Hemoglobin and hematocrit were mildly reduced but not significantly different across groups. In contrast, MCV and MCH were significantly increased at both time points in patients with AN, indicating macrocytosis, with a modest but significant reduction in MCH from T0 to T1. MCHC was consistently decreased in patients with AN, suggesting hypochromia.

**Table 2:** Blood counts obtained from the blood tests of patients with AN and HC at both T0 and T1.

	HC (n = 26) *(*±*SD)*	AN T0 (n = 35) *(*±*SD)*	AN T1 (n = 20) *(*±*SD)*	AN T0 vs HC *p* value	AN T1 vs HC *p* value	AN T0 vs AN T1 *p* value
**Red Blood Cells (RBC)** 3.9–5.1 mil/mm³	4,69 (0,38)	4,37 (0,37)	4,43 (0,45)	0,0015**	0,0397*	0,7788
**Hemoglobin** 12–14 g/dl	13,77 (1,03)	13,42 (0,65)	13,42 (0,87)	0,1515	0,1970	0,8328
**Hematocrit** 37–46%	41,27 (2,48)	40,43 (2,07)	40,61 (2,63)	0,1382	0,4364	0,6303
**Mean Corpuscular Volume (MCV)** 84–98 fL	88,17 (4,10)	92,61 (4,69)	92,16 (5,70)	0,0002***	0,0083**	0,8575
**Mean Corpuscular Hemoglobin Concentration (MCHC)** 32–35.5 g/dl	33,38 (0,97)	30,89 (2,10)	30,43 (2,32)	<0,0001***	<0,0001***	0,9779
**Mean Corpuscular Hemoglobin (MCH)** 28–33 pg	29,48 (1,82)	33,28 (1,02)	32,68 (1,03)	<0,0001***	<0,0001***	0,0554
**Platelets** 160–350 /mm^3^	276,08 (70,46)	220,60 (20,33)	219,90 (32,04)	0,0022**	0,2252	0,2842
**Basophil %** 0–5.2%	0,73 (0,37)	2,14 (2,21)	2,75 (2,72)	0,2050	0,4159	0,8652
**Basophils (Absolute Count)** 0–0.1 /mm^3^	0,05 (0,02)	0,04 (0,01)	0,04 (0,02)	0,1409	0,7840	0,5484
**Eosinophil %** 0–1.4%	2,18 (1,77)	0,77 (0,29)	0,81 (0,29)	0,7636	0,2226	0,2118
**Eosinophils (Absolute Count)** 0–0.6/mm^3^	0,15 (0,14)	0,14 (0,12)	0,17 (0,17)	0,5161	0,1918	0,2290

The ranges shown beneath the parameters are for reference only and reflect the physiological intervals typically adopted in clinical settings. Results are presented as raw data ± SD. Unpaired Student’s t test (HC vs AN T0, AN T1); Paired t test (AN T0 vs AN T1).

Statistical significance is calculated at p < 0.05 (*); (**p < 0.01, ***p < 0.001). Detailed statistics are presented in Supplementary Table 4.

At T0, patients with AN show increased levels of plasma cortisol (Fig. 1l), and in a subpopulation of patients with AN representative of the entire cohort, we found reduced circulating, along with reduced abundances, of leukocytes (Fig. 1n), neutrophils (Fig. 1o), monocytes (Fig. 1p) and lymphocytes (Fig. 1q). This phenotype overall suggests a condition of leukopenia, which fits with the observation of a reduced quantity of CD34+/CD45dim cells (a widely used indicator of hematopoietic stem cells (HSCs) density in the bone marrow [42]) in anorectic patients versus HC as well (Fig. 1m). This observation was further corroborated by the reduced plasma levels of monocyte and the granulocyte-monocyte colony stimulating factors M-CSF and GM-CSF in AN compared to HC (Fig. 1r). Of note, we observed an increased CD62L membrane expression in all the circulating leukocytes of AN as MFI (Mean Fluorescence Intensity) versus to HC (Fig. 1s-v), but this was not reflected by significant changes in the plasma levels of a series of cytokines and inflammatory mediators, including MAD-CAM, E-selectin TNFα, CX3CL1 and CCL2, which were comparable between subjects with AN and HC. Together these observations suggest an altered distribution of the immune cells landscape between bone marrow and the blood, and the derailment of compensatory immune-inflammatory patrolling in anorectic conditions, potentially indicating an innate immune cell activation and recruitment at site of inflammation.

### Women with AN continue to exhibit hypercortisolism after 1 year of follow up and only recovered women boosted monocyte recruitment

**Fig. 2:**
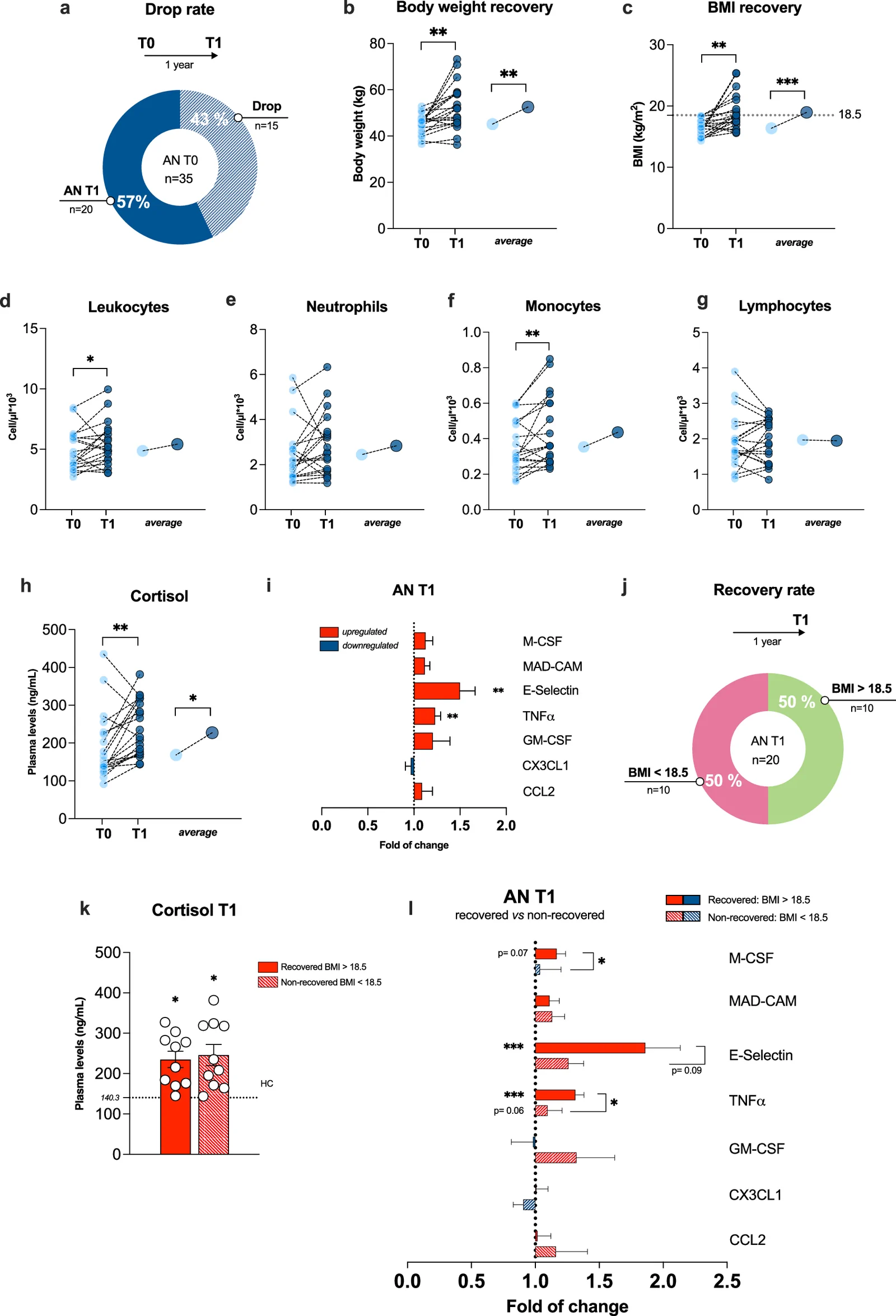
Non-recovered AN subjects show increased cytokines plasma levels and monocytes recruitment after 1 year of follow-up. Drop rate of patients from the initial AN cohort after 1 year (T1) of follow-up **a**. Body weight (BW, **b**) and body mass index (BMI, **c**) of subjects with AN at T1 (n = 20) which remained under evaluation. Blood cell counts of leukocytes **d**, neutrophils **e**, monocytes **f**, lymphocytes **g** and cortisol levels **h** of T1 subjects with AN. Levels of circulating cytokines and chemokines in AN T1 subjects vs AN T0 **i**. Rate of recovered subjects **j**, cortisol levels **k** and circulating cytokines and chemokines in recovered vs non-recovered patients with AN at T1 **l**. Cytokines levels measured in the plasma of HC and subjects with AN T1 are expressed as fold of changes (**i**, **l**). Paired Student’s t test (T0 vs T1, in panels **b**-**h**, left), Unpaired Student’s t test (average, in panels **b-h** right, **I, k**), Mixed effect one-way ANOVA **k**. Statistical significance is calculated at p < 0,05. ^*^p < 0,05, ^**^p < 0,01, ^***^p < 0001. Detailed statistics are presented in Supplementary Table 6.

After one year of follow-up (T1) from the initial clinical evaluation, we recorded a 43% drop-out rate (Fig. 2a), meaning that only 57% of the patients enrolled at T0 remained in care (*n* = 20), while the others discontinued the service (*n* = 15). The patients at T1 showed a significant increase in both the BW (Fig. 2b) and BMI (Fig. 2c). At cellular level only leukocytes and monocytes raised significantly their counts compared to the T0 (Fig. 2d, f), while neutrophils and lymphocytes did not change in respect to T0 (Fig. 2e, g). Of note, patients showed even higher cortisol levels in respect to the T0 (Fig. 2h), and unexpectedly elevated levels of circulating E-selectin and TNFα (Fig. 2i), which were unaltered at T0. No further changes were detected for the other molecules.

Considering the heterogeneous clinical progression observed among all enrolled patients after one year, we decided to further stratify the T1 cohort based on their BW. This stratification allowed us to classify individuals as either BMI-recovered (BMI > 18,5) or BMI-non-recovered (BMI < 18,5). We found that 50% of patients did not recover, maintaining a low BMI, while the other 50% successfully restored their BW, surpassing the 18.5 threshold (Fig. 2j). Regardless of their BW condition, all patients affected by AN maintained elevated plasma levels of cortisol (Fig. 2k), but the increase in E-selectin and TNFα mentioned above were selectively attributable to the subjects classified as BMI-recovered. Moreover, M-CSF levels were significantly increased only in BMI-recovered individuals (Fig. 2l).

### Female rats developing the AN phenotype show compulsive hyperactivity

**Fig. 3:**
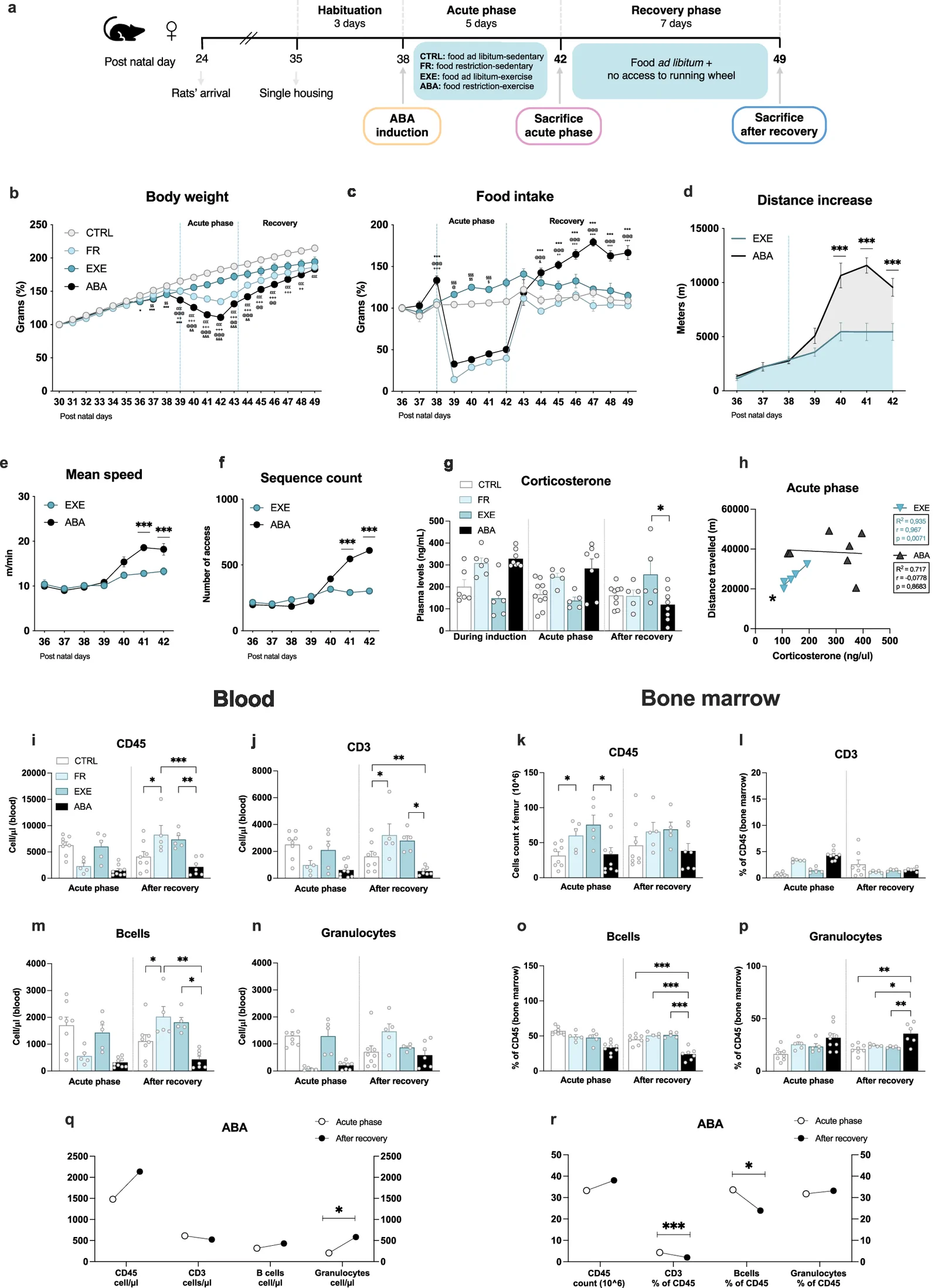
ABA rats alter their immune cell cellularity boosting innate immune response after a period of recovery. Experimental paradigm **a**. Average daily body weight (BW, **b**), food intake (FI, **c**) of CTRL, FR, EXE, and ABA rats. Distance increase **d**, mean speed **e**, and sequence counts **f** in EXE and ABA rats. Results are presented as the mean of 8–10 rat/group ± SEM. ^$^ p < 0.05, ^$$^ p < 0.01 CTRL vs EXE, ^*^ CTRL vs ABA, ^&^ FR vs EXE, ^@^ FR vs ABA, ^+^ EXE vs ABA, ^£^ CTRL vs FR and ABA, ^§§§^ p < 0.001 CTRL vs FR and ABA, EXE vs FR vs ABA. Corticosterone levels **g** measured in the plasma of CTRL, FR, EXE, and ABA rats during the induction of the AN phenotype (P40), at the acute phase (P42) and after recovery (P49). Pearson’s product–moment correlation **r** analyses between corticosterone levels at P42 and total distance travelled during the phenotype induction in ABA and EXE rats **h**. CD45, CD3, B cells, and granulocytes levels measured in blood **i, j, m, n** and bone marrow **k, l, o, p** in CTRL, FR, EXE, and ABA rats at both timepoints. CD45, CD3, B cells, and granulocytes levels measured in ABA rats at the acute phase and after recovery in blood **q** and bone marrow **r**. Data are expressed as percentages of CD45^+^cells. Results are presented as percentage of the mean ± SEM. ^*^p < 0,05, ^**^p < 0,01, ^***^p < 0001. CTRL control, FR food-restricted, EXE exercise, ABA activity-based anorexia. Detailed statistics are presented in Supplementary Tables 7–11.

In line with our previous findings [39, 43] we demonstrated that only the animals exposed to the combination of food restriction and wheel running (ABA) developed the AN phenotype. Indeed, at P42 (acute manifestation of the AN phenotype), weight loss of ABA rats was significantly greater than FR, despite similar food intake (Fig. 3b, c), highlighting the role of exercise in accelerating BW loss. ABA rats showed a significant increase in distance traveled (Fig. 3d), mean speed (Fig. 3e), and number of sequence counts (Fig. 3f) with respect to the only exercise (EXE) group. During the refeeding period (P42-P49), ABA rats showed a significantly higher food intake compared to the food-restricted (FR) group (Fig. 3c); nonetheless, at the end of the recovery period (P49) they did not reach the BW of control animals (CTRL) (Fig. 3b), indicating that they may need more time to achieve a full recovery.

Consistent with our previous findings [41], ABA rats showed elevated corticosterone levels during the induction of the AN phenotype (P40) that remained elevated at the acute phase (P42, Fig. 3g), an effect that appears to be primarily driven by food restriction, as the food restriction x exercise interaction was not significant in the two-way ANOVA. Of note, after recovery, corticosterone levels were reduced in ABA rats compared to the EXE group (Fig. 3g). Interestingly, the levels of corticosterone measured at P42 in the EXE group resulted to be positively correlated with the distance travelled (m), a pattern not observed in the ABA group (Fig. 3h). This suggests that moderate levels of exercise, in healthy conditions, are associated with circulating corticosterone levels, whereas in ABA rats such correlation is lost, likely due to the elevated corticosterone levels [44].

### ABA rats show altered peripheral immune cells profile at the acute phase enhancing bone marrow granulocytes production after recovery

Given the altered immunophenotype observed in subjects with AN and the relevant interplay between GCs and the immune system, we characterized immune cells in the ABA rats. In the blood, we observed a main effect of food restriction in reducing the whole pool of CD45+ leukocytes, and out of them, of CD3+ lymphocytes, B cells and granulocytes (Fig. 3i, j, m, n) in the ABA rats at P42, while these were not affected by EXE only. After recovery, we observed an increase in CD45+, CD3+ and B cells in FR rats, while a significant reduction in the ABA group (Fig. 3i, j, m, n).

In the bone marrow, at P42 we observed only an increase of the whole pool of CD45+ cells in EXE (Fig. 3k). At P49, ABA rats show a significant reduction in B cells counts (Fig. 3o), and an increase in granulocytes (Fig. 3p), further pointing to persistently altered immune profile even after recovery.

Interestingly, when comparing selectively the ABA groups in a before and after analysis (P42-49), we reveal a significant increase in circulating granulocytes (Fig. 3q), together with reduced CD3+ and B cells in the bone marrow (Fig. 3r). These effects suggest a compensatory increase of granulocytes in the recovery phase in the bone marrow that is reflected in their increased circulating levels compared to the acute phase.

### ABA rats in the acute phase show altered glucocorticoid receptor nuclear content and GR-responsive gene expression in the ventral hippocampus

After GCs secretion, circulating corticosterone can bind to its GR receptor, initiating a genomic signaling cascade that modulates gene expression either through direct binding to glucocorticoid response elements (GREs) in promoter regions or via interactions with other transcription factors. In this context, we assessed the expression of key GR-related targets, including *Gilz* and *Sgk1*, as prototypical GR-responsive genes, the receptor isoforms *GRα* (functionally active) and *GRβ* (regulatory), and *Fkbp5*, a critical modulator of GR sensitivity and feedback regulation, to better characterize GR pathway activation and dynamics.

**Fig. 4:**
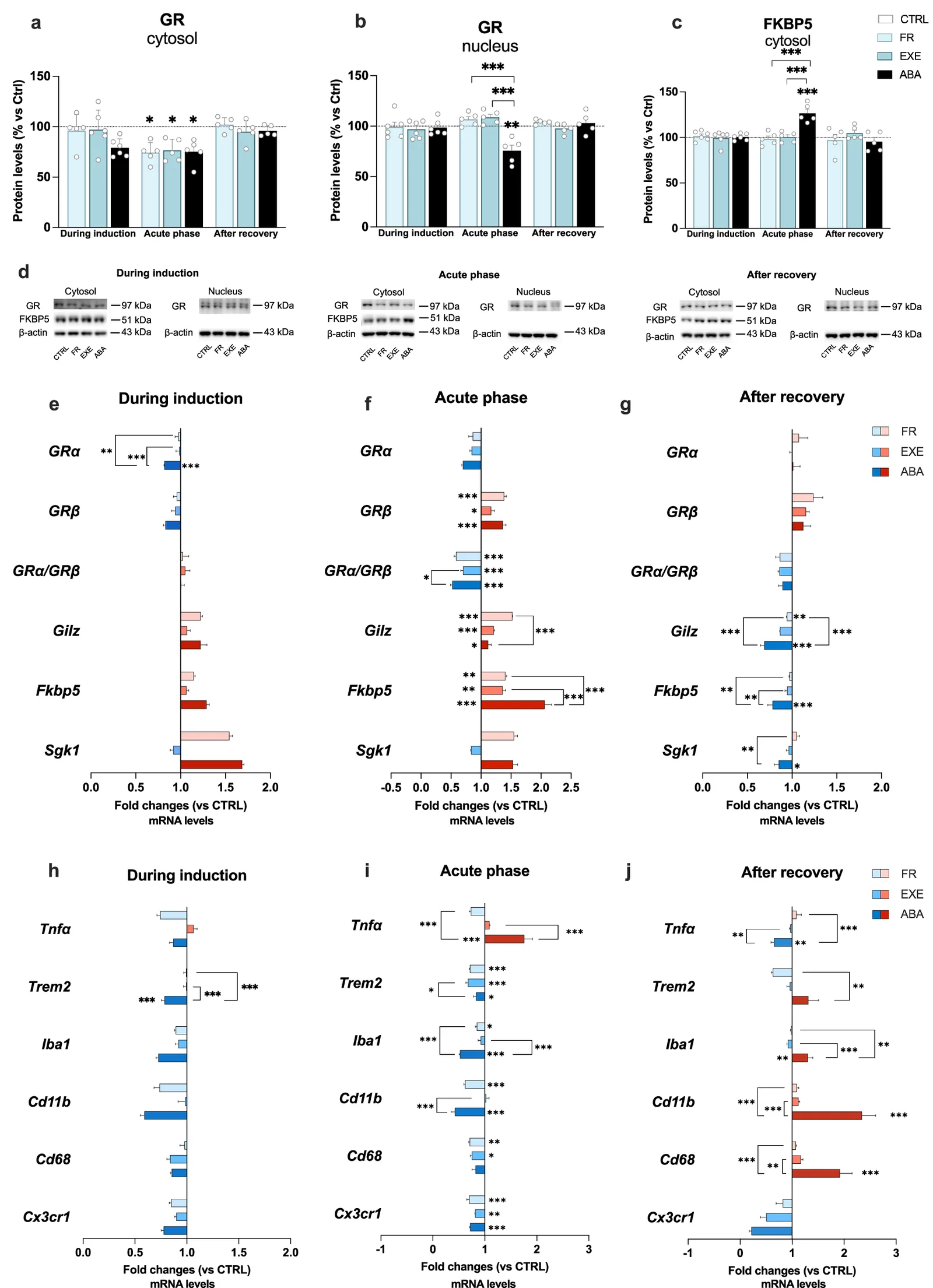
ABA induction alters nuclear GR protein content and GR-responsive genes expression promoting a pro-phagocyting profile of microglia markers in the ventral hippocampus after recovery. Protein levels of Glucocorticoid receptor (GR) measured in the cytosolic **a** and nuclear **b** fraction, and of FKBP5 in the cytosol **c** of the ventral hippocampus of CTRL, FR, EXE, and ABA rats during the induction of the AN phenotype, at the acute phase and after recovery. Data are expressed as percentages of CTRL (dashed line). Results are presented in % as the mean of 5–6 rat/group ± SEM (**a-c**). ^*^p < 0,05, ^**^p < 0,01, ^***^p < 0001 vs CTRL. Panel **d** shows representative cropped immunoblots of GR receptor, FKBP5 and the internal standard β-actin. Gene expression of *GRα*, *GRβ*, *Gilz, Fkbp5* and *Sgk1* measured in CTRL, FR, EXE, and ABA rats during the induction of the AN phenotype **e** at the acute phase **f** and after a week of bodyweight recovery **g**. Gene expression of *Tnfα*, *Trem2*, *Iba1*, *Cd11b*, *Cd68*, and *Cx3cr1* measured in CTRL, FR, EXE, and ABA rats during the induction of the AN phenotype **h** at the acute phase **i** and after recovery **j**. Results (**e-j**) are expressed as fold changes. ^*^p < 0,05, ^**^p < 0,01, ^***^p < 0001 vs CTRL. CTRL control, FR food-restricted, EXE exercise, ABA activity-based anorexia. Detailed statistics are presented in Supplementary Tables 12–13.

At P42, GR protein expression in the cytosolic fraction was significantly reduced in the three experimental groups tested (Fig. 4a), while in the nucleus the GR content was significantly reduced only in ABA rats (Fig. 4b). FKBP5, co-chaperone responsible for inhibiting GR translocation in the nucleus, was significantly increased only in ABA rats at P42 (Fig. 4c). No changes were detected either at P40, during AN induction, or at P49, following the recovery period in both cytosolic and nuclear fractions (Fig. 4a-c). Looking at the gene expression of *GRα*, *GRβ, Gilz*, *Sgk1* and *Fkbp5*, we observed a time- and condition-dependent pattern of expression. At P40, *GRα* was significantly downregulated only in ABA rats and *GRβ*showed a similar trend although not significant. Conversely, *Gilz* and *Sgk1* expression was upregulated in both ABA and FR animals, due to a main effect of the food restriction, indeed no food restriction x exercise interaction was detected. *Fkbp5*, instead, was unaltered showing only a tendency towards an increase in ABA rats (Fig. 4e). At the acute phase, *GRα*presented a slight reduction, yet not significant, while *GRβ*instead was strongly upregulated in all experimental groups vs CTRL, thus resulting in a significant decrease of the *GRα /GRβ* ratio (Fig. 4f). *Gilz* mRNA levels were increased in FR, EXE and ABA animals in respect to CTRL (Fig. 4f). Of note, *Fkbp5* was strongly upregulated only in ABA rats (Fig. 4f). After recovery, we did not detect any significant changes in *GRα*or* GRβ*; *Gilz* was significantly reduced in EXE and ABA vs CTRL, and of note, the reduction observed in ABA was even greater than FR and EXE (Fig. 4g). *Fkbp5* gene expression levels were downregulated only in ABA rats (Fig. 4f).

### The AN phenotype in female rats alters the microglial gene expression pattern showing a pro-phagocytic profile after a period of recovery

Considering the broader modulatory effects of GR signaling on neuroimmune function, we assessed the gene expression of selected markers reflecting microglial activation and functional state, which can be directly or indirectly regulated by GR, thus providing additional insight into GR-related modulation of microglial and inflammatory pathways. We assessed Trem2, since it is associated with phagocytic activity and microglial survival, Iba1 and Cd11b, as markers of microglial activation, and Cx3cr1, involved in neuron–microglia communication, along with the pro-inflammatory cytokine Tnfα.

During the induction of the AN phenotype we did not observe many changes, with the only exception of a significant downregulation of *Trem2* in ABA rats (Fig. 4h). However, at P42, we observed an overall downregulation of microglial markers, such as *Trem2, Iba1*, *Cd11b* and *Cx3cr1* in ABA rats vs CTRL, FR or EXE groups, except for *Tnfα,*which is the only one significantly upregulated in respect to the other groups (Fig. 4i). Interestingly, at P49, ABA rats showed a completely different picture which revealed a significant reduction of *Tnfα*, together with an increase in all the other markers with the only exception of *Cx3cr1*, which did not show significant changes (Fig. 4j). Together these results suggest that the microglia are shifting their phenotype from a pro-inflammatory not surveillant state, in the AN acute phase, towards a more pronounced pro-phagocytic tissue‑remodeling profile after a period of recovery.

### Treatment with RU486 dampens the hyperactive compulsive behaviors in ABA rats and reduced CD3+ cells production from the bone marrow

**Fig. 5:**
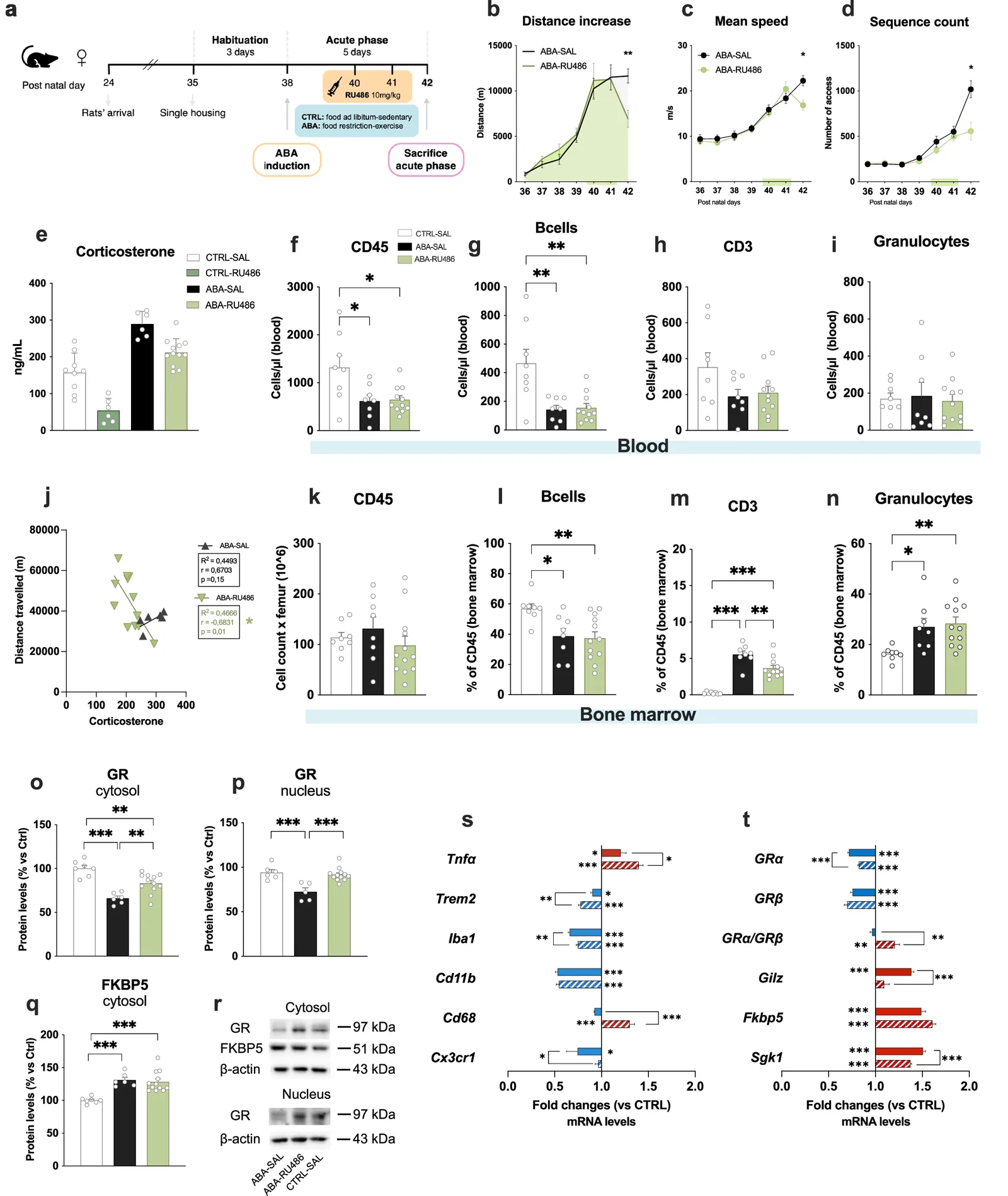
GR antagonist dampens compulsive hyperactive behavior in ABA rats, restores GR protein content and reshapes microglia phenotype. Experimental paradigm **a**. Distance increase **b**, mean speed **c**, and sequence counts **d** measured in ABA rats treated with saline (ABA-SAL) and with the GR antagonist RU486 (ABA-RU486). Corticosterone plasma levels **e** measured in CTRL-SAL/RU486, ABA-SAL/RU486 rats at the acute phase. CD45, CD3, B cells, and granulocytes levels measured in blood **f, g,** **h,** **i** and bone marrow **k,** **l,** **m,** **n** in CTRL-SAL, ABA-SAL/RU486 rats. Data are expressed as percentages of CD45^+^cells. Pearson’s product–moment correlation **r** analyses between corticosterone plasma levels and the distance travelled during the phenotype induction in ABA-SAL and ABA-RU486 **j**. GR protein levels measured in the cytosolic **o** and nuclear **p** fraction, and of FKBP5 in the cytosol **q** of the ventral hippocampus in CTRL-SAL, ABA-SAL and ABA-RU486 rats at the acute phase. Panel **r** represents immunoblots of GR receptor and the internal standard β-actin. Gene expression levels of *GRα, GRβ*, *Gilz, Fkbp5*, *Sgk1* **s**, and of *Tnfα*, *Trem2*, *Iba1*, *Cd11b*, *Cd68*, and *Cx3cr1* measured in CTRL-SAL, ABA-SAL/RU486 rats **t**. Results are expressed as mean or fold changes vs CTRL-SAL and presented as the mean of 6–12 rats/group ± SEM. ^*^p < 0,05, ^**^p < 0,01, ^***^p < 0001 vs CTRL-SAL. CTRL-SAL: control + saline treatment, ABA-SAL: activity-based anorexia + saline treatment, ABA-RU486: activity-based anorexia + RU486. Detailed statistics are presented in Supplementary Tables 14–19.

To verify the implication of corticosterone in driving hyperactive behaviors during the ABA paradigm, we treated ABA rats with 10 mg/kg of RU486, a non-selective GR antagonist (Fig. 5a). RU486 significantly reduced wheel distance travelled (Fig. 5b), mean speed (Fig. 5c) and sequence counts (Fig. 5d) of ABA rats at P42, while it did not change the number of wheel accesses to perform brief exercise sequences (Supplementary Fig. 1), an index of playful behavior. As a proof of concept, RU486 lowered the significantly higher levels of circulating corticosterone in both ABA and CTRL animals (Fig. 5e). Of note, even though the levels of corticosterone in ABA rats did not correlate with the distance travelled on the wheel, after the RU486 treatment, we observed a negative and significant correlation highlighting that higher distance travelled correspond to lower corticosterone levels (Fig. 5j).

Even though the RU486 treatment was not effective in modulating white blood cells neither in the blood nor in the bone marrow of ABA rats (Fig. 5f-i), such treatment was able to reduce only the CD3+ cells in the bone marrow of ABA rats (Fik. 5n), which were indeed significantly higher in ABA-saline in respect to CTRL. This effect may suggest that RU486 has not major effect on peripheral immune cells since it impacted only on T cells in the bone marrow, while it does not affect leukocytes or circulating cells.

### RU486 restored GR levels and reshaped the microglial gene expression profile in the ventral hippocampus of ABA rats

Similarly to what observed in the periphery, treatment with RU486 was only partially effective also in the ventral hippocampus. Indeed, even though it moderately restored the cytosolic levels of GR (Fig. 5o), it completely restored the GR in the nucleus of ABA rats (Fig. 5p) at the levels of CTRL, while FKBP5 protein levels were unaltered (Fig. 5q). In line with that, the modulation with RU486 was able to significantly reduce *Gilz* and *Sgk1* gene expression which were upregulated by the ABA condition per sé, and to dampen the reduction of *GRα* expression which was significantly downregulated in ABA rats (Fig. 5s). However, even though *GRβ* levels were not modulated by the treatment per sé, the *GRα /GRβ* ratio was significantly increased, thus pointing towards an enhanced transcription of the *GRα*over the *GRβ*(Fig. 5s). *Fkbp5* gene expression levels were not modulated by the treatment (Fig. 5s). In addition, looking at the microglia markers, even though RU486 further enhanced the levels of *Tnfα*and reduced *Trem2*, it narrowed *Iba1* and *Cx3cr1* downregulation, and markedly increased the levels of *Cd68* in respect to ABA-saline and CTRL (Fig. 5t), thus suggesting that RU486 may be able, at least partially, to reshape the microglia phenotype from a pro-inflammatory to a pro-phagocyting/surveillance state.

## Discussion

AN lacks reliable biomarkers for disease progression, which hamper the development of personalized treatments and poses significant challenges for long-term clinical management. To address this gap, we integrated data from both patients with AN and the ABA preclinical model. We show that AN is characterized by profound dysregulation in the HPA axis-mediated stress response, accompanied by significant adaptations in both peripheral and central immune systems that persist despite clinical remission, thereby providing a putative molecular mechanism underlying “latent” disease maintenance.

Our cohort of women diagnosed with AN scored significantly higher on the EED-19 questionnaire alongside the classical symptoms of food avoidance and body weight loss [45, 46]. Although hyperactivity is not consistently elevated across all subjects with AN [47, 48], the heightened self-reported exercise engagement in daily life found here was consistent with our previous observation in a larger cohort [49]. Moreover, qualitative analysis of their responses revealed a unique compulsive attitude toward physical activity, along with a loss of the perception of exercise as positive and healthy, an aspect that was instead preserved only in HC. Except for a few studies that associate hyperactivity with hormonal dysfunctions in AN [50, 51], no evidence of mechanisms directly linking the physical exercise with disease outcomes or progression are available. We herein show that patients who compulsively engage in physical exercise exhibit elevated cortisol levels and marked leukopenia, consistent with previous findings in similar cohorts [52]. As a novelty from the current knowledge, underweight individuals with AN (at T0) also show reduced plasma levels of M-CSF and GM-CSF, which may reflect a broader impairment in myeloid lineage differentiation and activation, resulting in a weakened innate immune response [53], potentially driven by a hyperactivation of the HPA axis. This immunosuppressive profile may contribute not only to increased vulnerability to infections, but also to altered neuroimmune communication ultimately sustaining the pathological state and impeding full recovery. We herein add a piece of information demonstrating that despite white blood cells were reduced in these subjects, they were more activated as shown by increased CD62L (L-selectin), which plays a crucial role in guiding leukocytes to lymphoid tissues and sites of inflammation [54]. Its upregulation could reflect a compensatory mechanism attempting to counterbalance the impaired myeloid differentiation and activation caused by reduced M-CSF and GM-CSF levels. This scenario may help to explain why patients with AN appear to be less susceptible to infections, despite their leukopenia. However, in some cases, persistent high CD62L expression may also contribute to immune cell sequestration within lymphoid organs, potentially limiting effective peripheral immune surveillance [55].

After one year of follow-up (T1), considering that the high attrition rate is in line with the well-known longitudinal follow-up data in this population [56], the group of subjects with AN showed an increase in BW and BMI, as well as in leukocyte counts, particularly monocytes, in respect to AN T0. Even though they still exhibited hypercortisolemia, which is further increased compared to patients with AN at T0, their cytokine profile changed significantly from baseline. They showed elevated levels of TNFα, highlighting a situation of peripheral inflammation which was absent at T0, and of E-selectin, an inducible adhesion molecule expressed on activated endothelial cells in response to inflammatory cytokines that facilitates leukocyte rolling and recruitment at inflamed tissues. Given this profile, to better dissect the cellular fingerprints of the disease, we calculated a recovery rate to stratify the AN T1 population into subjects who regained BW and those who did not in one year. Notably, we found that the specific increases in circulating TNFα and E-selectin were confined to the subgroup of patients classified as “recovered,” defined by reaching a BMI greater than 18,5 kg/m². Moreover, those subjects were also characterized by elevated M-CSF, fundamental for monocytes/macrophages differentiation, which indicates a push toward innate immune activation. These effects were particularly intriguing because cortisol levels remained elevated indiscriminately in both recovered and non-recovered subjects. This further supports the notion that a hyperactivation of the HPA axis may result in the inability to maintain a homeostatic fluctuation of GCs and an impairment in GR function, which in turn may induce long-lasting changes in the immune response due to the loss of the physiological immunosuppressive effect of cortisol. This may indicate a potential desensitization of immune cells to GC signaling, also known as “glucocorticoid resistance”, a phenomenon reported in other chronic stress and inflammatory conditions, such as major depression disorder [57–59].

To gain deeper insights into the mechanisms underlying these findings in human subjects, we moved to the rodent ABA model. When ABA rats reach the acute manifestation of the AN phenotype, they show elevated corticosterone levels, as recently demonstrated by our group [41], an effect likely driven by acute starvation. However, ABA rats display a general reduction in circulating white blood cells, a condition that persists even after BW recovery, akin to FR animals that instead recover physiological counts. Interestingly, analysis of the bone marrow revealed a trend toward increased in granulocytes, alongside a reduction in B cell populations, which turned out significant after recovery. Indeed, when comparing ABA animals before and after recovery, we observed a marked increase in circulating granulocytes accompanied by a sustained reduction in bone marrow-derived B cells, suggesting a lasting skewing toward innate immune activation.

Given the persistence of peripheral immune alterations after recovery, we next examined whether similar changes could be detected centrally, focusing on the ventral hippocampus as a region sensitive to stress and crucial for emotional regulation. To evaluate GR expression and its transcriptional activity, we measured the temporal profiles of GR across subcellular fractions, its co-chaperone FK506-binding protein 51 (FKBP5), GR-responsive genes and key microglial markers from two days after anorexia induction through the acute phase and up to seven days of BW recovery.

The induction of the AN phenotype downregulated both *GRα* and *GRβ* mRNA levels without affecting either the GR or FKBP5 protein expression or subcellular localization in ABA rats. At the acute phase, we observed a selective downregulation of *GRα* together with the upregulation of *GRβ* in ABA rats, paralleled by a reduction of GR in both cytosolic and nuclear fractions and an increase in FKBP5 in the cytosol. Given that *GRβ* modulates GR function by acting as a dominant negative inhibitor of *GRα*, thereby reducing GR responsiveness [60] and promoting glucocorticoid resistance [61], and given that FKBP5 acts as a negative regulator of GR activity delaying its nuclear translocation [62], it appears that these mechanisms might affect GR-dependent transcriptional activity thus contributing to the glucocorticoid resistance [63, 64]. *Gilz*, a canonical GR target gene, was significantly upregulated in the acute phase, indicating that despite lower GR protein levels, the receptor remained transcriptionally active. Interestingly, after recovery, despite both *GRα* and *GRβ* gene expression and GR or FKBP5 protein levels were restored to CTRLs levels, *Gilz* mRNA levels were significantly downregulated only in ABA rats, suggesting a persistent alteration in GR-dependent downstream transcriptional responsiveness.

Physiologically, active GR signaling exerts anti-inflammatory effects, including trans-repression of pro-inflammatory genes like *Tnfα* [65]. Notably, in the acute ABA condition we observed increased *Tnfα* expression, suggesting that GR trans-repressive function may be compromised [64]. Interestingly, after recovery, GR nuclear content was restored and *Tnfα* expression declined. Markers of microglial activation, *Iba1, Trem2*, and *Cd11b*, were downregulated during the acute phase despite elevated *Tnfα*, whereas after recovery, all these markers, together with *Cd68*, were upregulated. This pattern suggests a transition toward a more phagocytic, tissue-remodeling microglial phenotype occurring during the recovery phase, rather than representing a sustained pro-inflammatory response.

Thus, tracking the expression of GR-related markers in the ventral hippocampus over time reveals a complex but clear time-dependent transcriptional profile: initially latent during the induction phase, which becomes pronounced at the acute stage highlighting a decoupling between GR activation and its downstream transcriptional effects, which culminate in a rebound microglial response after recovery. One possible interpretation is that, during the acute phase, ABA rats develop signs of glucocorticoid resistance, which may in turn prime microglia to enter a phase of active remodeling during recovery, potentially in response to tissue damage or altered synaptic plasticity induced by the earlier acute phase. This may reflect a secondary phase of the disorder, regardless of the low body weight, yet significant from a neurobiological perspective. Accordingly, our previous findings reveal that ABA induction orchestrates hippocampal maladaptive structural and functional plasticity and long-lasting spatial memory deficits, paralleled by dysfunctional GR-mediated non genomic stress response [41].

Interestingly, pharmacological modulation of the GR system with RU486, a non-selective GR antagonist, during the induction of the AN phenotype (first injection at P40, i.e. initiation of the exercise escalation) significantly reduced compulsive hyperactivity in ABA rats, ameliorating one of the core symptoms of the disorder. As a proof of concept, RU486 reduced corticosterone levels in CTRL animals and, even though to a lesser extent, also in ABA rats. Although the treatment did not broadly alter immune cell composition, presumably due to its short duration, RU486 effectively restored GR nuclear localization in the ventral hippocampus of ABA rats, also modulating the balance between *GRα* and *GRβ* expression favoring the transcription of the *α* over the *β* isoform, and promoting an early shift in microglial phenotype during the acute phase, as indicated by a significant increase of *Cd68* gene expression. Notably, *Cd68* expression remained unchanged in untreated rats during the acute phase and became significantly upregulated only after recovery. The selective upregulation of *Cd68* together with *Tnfα*, in the absence of a broader activation of the other markers, may reflect a primed or surveilling microglial state characterized by heightened sensitivity but limited phagocytic engagement.

These findings highlight the existence of a miscommunication between the GC and immune systems suggesting that both patients with AN and ABA rats develop, to some extent, a condition of glucocorticoid resistance. This may help explaining some of the disorder most puzzling clinical features, such as the ability of patients with AN to perpetuate aberrant behaviors to maintain weight loss and enduring exercise for years despite prolonged and severe malnutrition. Moreover, we reveal a specific and persistent molecular signature of AN in patients who have achieved BMI recovery, further supporting the notion that full recovery from AN extends beyond body weight restoration. These long-lasting biological alterations, as the prolonged elevation of cortisol and the immune cell adaptation, may contribute to disease maintenance and represent a critical risk factor for relapse. In addition to these insights, our work may indeed represent a step forward in clinical practice by identifying stage-dependent biomarkers, challenging the prevailing reliance on body weight restoration as the primary remission criterion.

However, we acknowledge that this work presents limitations. The sample size is relatively small, which, combined with a considerable drop-out rate, an issue unfortunately intrinsic to this pathology, limits statistical power and affects the robustness of stratification measures at T1, especially given that the analyses start from an already limited number of participants. Self-reported assessments (EED-19) may be subject to response biases, and this should be considered when interpreting the results. Moreover, we acknowledge that, despite our efforts in excluding pharmacological treatments, variability in medication use cannot be entirely excluded, even though the prescription had been initiated recently. The study also lacks a sex-based comparative analysis, an increasingly critical aspect of AN research, particularly considering the rising prevalence of the disorder among male subjects. This limitation hampers the identification of potential sex-specific differences in both pathophysiology and treatment responsiveness. Additionally, although the pharmacological intervention exhibited partial efficacy in the animal model, the absence of long-term follow-up data prevents any definitive conclusions regarding its sustained impact. While we hypothesize that the observed alterations may underlie mechanisms of relapse vulnerability, this remains speculative, as the study did not include targeted experimental validation to support this assumption. To address these limitations, ongoing work is focused on incorporating sex as a biological variable, extending the duration of follow-up, increasing sample sizes where possible, and designing experiments specifically aimed at validating the proposed relapse-related mechanisms.

## Methods

### Subjects

In total, 35 subjects with AN and 27 healthy controls (HC) were recruited for this study. Patients were recruited at the Eating Disorders Clinic of the ASST Santi Paolo and Carlo Hospital (Milan, Italy) between 2021 and 2023. All participants were outpatients and sex matched.

The diagnosis of AN was established according to the latest available criteria from the Diagnostic and statistical manual of mental disorders (DSM-5) [3]. Psychiatric and eating disorder psychopathology were assessed by experienced clinicians using psychometric evaluation supported by the Symptoms Checklist-90 (SCL-90) [66] and the Eating Disorder Inventory-3 (EDI-3) [67].

All participants and their legal guardians in the case of minors provided written informed consent. The study received approval from the Milan Area 1 Ethical Committee (Number of approval: 0052445, 03/12/2020) and was carried out in accordance with the Declaration of Helsinki.

### Human study design and selection criteria for human subject recruitment

To avoid excessive heterogeneity of the AN cohort that might increase the risk of biases, inclusion criteria for this population were: (1) time of illness from 12 to 36 months and (2) BMI < 18,5; and exclusion criteria were: (1) organic brain syndrome, (2) schizophrenia, (3) substance use disorder, (4) bipolar disorder, (5) bulimia nervosa or (6) binge-eating disorder. Thirty-five of the 50 recruited patients satisfied all criteria and were therefore included in the analysis. Inclusion criteria for the control sample were (1) a normal body weight (BMI 18,5–25), and the exclusion criteria were (1) presence of known chronic or autoimmune diseases, (2) diagnosis of any psychiatric disorder and (3) any chronic medication apart from birth control pills and psychoactive compounds. Subjects receiving psychopharmacological treatment were still included in the cohort of 35. This choice reflects current clinical reality, in which a substantial proportion of patients with AN are prescribed psychotropic agents for comorbid symptoms, and nearly all major classes (antidepressants, antipsychotics, benzodiazepines) have been tried in AN despite the lack of robust, disorder-specific efficacy data. Specifically, 8 individuals treated with antidepressants (AD), 1 with a combination of AD+benzodiazepines (BDZ), 1 with antipsychotics (AP) + BDZ, 1 with AP + AD, and 1 with BDZ.

All subjects diagnosed with AN were evaluated for biometric parameters and underwent blood sampling at two time points: at the first contact with the clinic (T0), and one year after the first evaluation (T1). HC underwent biometric evaluation and blood sampling only once, at baseline. Blood samples were collected at 9:00 AM after an overnight fast by professional nurses, following the standard protocols of the ASST Santi Paolo and Carlo Hospital.

As mentioned above, in addition to the SCL-90 and EDI-3, at T0 and T1 all the patients were requested to answer the Exercise and Eating Disorder 19 (EED19) questionnaire, a validated self-reported survey consisting of 19 items covering three areas related to the quality and perception of physical exercise: compulsive exercise, healthy exercise combined with awareness of body signals and exercise to control body weight and figure [68].

Given the heterogeneous clinical progression observed among patients at the one-year follow-up (T1), participants were further stratified based on body weight (BW). Recovery status was operationally defined using BMI as the sole criterion, adopting the threshold of 18.5 according to the WHO (World Health Organization) guidelines, which distinguishes underweight (BMI < 18.5) from normal weight (BMI ≥ 18.5). Accordingly, individuals were classified as BMI-recovered (BMI ≥ 18.5) or BMI-non-recovered (BMI < 18.5).

For each time point (T0-T1), all blood samples were processed at the Department of Biomolecular and Pharmacological Sciences “Rodolfo Paoletti” (DiSFeB, University of Milan, Italy) for flow cytometry and plasma isolation.

To establish the representativeness of the AN subpopulation (*n* = 7) used for the abovementioned analysis of circulating abundances of leukocytes (Fig. 1n-q) and CD62L membrane expression (Fig. 1s-v), relative to the total cohort (*n* = 35), baseline characteristics were compared. No significant differences were found across all key variables, including age, body weight, BMI, cortisol levels, and EED-19 scores.

### Animals and housing

Adolescent female Sprague-Dawley rats were purchased from Charles River (Calco, Italy). Upon their arrival animals were housed in groups of four rats per cage, with a reversed 12 h light/dark cycle (light on/off: 9.30 pm/9.30 am), under standard conditions of temperature (21 ± 1 °C) and humidity (50–60%). They were fed with standard rat chow (ssniff Spezialdiäten GmbH, Soest, Germany) and tap water *ad libitum*. A maximum of two female siblings was taken from each litter to reduce “litter effect” [69].

All the procedures were conducted at the DiSFeB, University of Milan, and performed according to the principles set out in the following laws and policies governing the care and use of laboratory animals: Italian Governing Law (D.lgs 26/2014; Authorization n.19/2008-A issued March 6, 2008, by Ministry of Health); the NIH Guide for the Care and Use of Laboratory Animals (2011 edition) and EU directives and guidelines (EEC Council Directive 2010/63/UE). Animal use was approved by the Italian Ministry of Health (#861-2023-PR). All efforts were made to minimize animal suffering and to employ the lowest number of animals: for ethical reasons, activity-based anorexia (ABA) rats were not allowed to lose more than 25% of their initial body weight. The experiments have been reported in compliance with the ARRIVE guidelines.

### Experimental design

*Experiment 1:* female rats arrived at postnatal day (P) 28, were group-housed, and acclimated for 7 days with food and water ad libitum (Fig. 3a). At P35, animals were individually housed and assigned to four groups: control (CTRL; sedentary, food ad libitum), food-restricted (FR; sedentary, 2 h/day food access), exercise (EXE; running wheel, food ad libitum), and activity-based anorexia (ABA; running wheel, 2 h/day food access). No randomization methods were used to allocate subjects in the study; the animals were arbitrarily divided by chance into the experimental groups. EXE and ABA animals were housed in activity cages equipped with running wheels. From P38 onward, FR and ABA rats had food access limited to 2 h/day (10:30–12:30), during which wheels were blocked. Body weight, food intake, and wheel activity were monitored daily from P35 until the end of the experiment. Animals were sacrificed at P40 (phenotype induction), P42 (acute phase), or P49 (after one week of body weight recovery under ad libitum feeding and sedentary conditions).

*Experiment 2:* a second cohort of female rats was singly housed at P35 and assigned to CTRL-SAL, CTRL-RU486, ABA-SAL, or ABA-RU486 groups (Fig. 5a). RU486 (10 mg/kg) or saline was administered intraperitoneally twice daily from P40 to P42.

All animals were sacrificed at P42. Hippocampi were dissected, frozen, and stored at −80 °C, and trunk blood was collected for subsequent analyses.

### Drug

RU486 C29H35NO2 (M8046; Sigma-Aldrich Company Ltd) was suspended in DMSO 25% and saline 0.9%.

### Flow cytometry

The immunophenotyping was performed on blood for both human subjects and rodents, while for rats also on the single-cell suspensions obtained from the bone marrow.

The blood was incubated with the antibody mix for 30 min at 4 °C and then fixed and lysed with Fix/Lyse 1× solution (eBioscience, San Diego, CA, USA) following the manufacturer’s instructions. To obtain a cell suspension from the bone marrow we used one femur per rat. Bones extremities were removed and placed in 15 ml tubes in PBS. Afterwards samples were rapidly centrifuged for 10 seconds at 16000 rcf to recover the bone marrow and red blood cells were lysed with the RCB lysis buffer (eBioscience). The cells were then counted, and 106 cells were stained with the antibody mix for the flow cytometry immunophenotyping at 4 °C for 30 min. The washes and the final resuspension for acquisition were performed using PBS with FBS 2% and EDTA 2 mM. The sample acquisition was performed with the LSRFortessa X-20 (BD) and the data analyzed with the Novoexpress software (version 1.6.0, Agilent, Santa Clara, CA, USA). The full list of antibodies and conditions is provided in Supplementary Table 1, and the gating strategy is represented in Supplementary Figs. 6–7.

### ELLA

Circulating protein levels in plasma samples of AN patients and HC have been detected with the multiplexing Ella™ Automated ELISA (Bio-techne, 614 McKinley Place NE, Minneapolis, MN 55413 USA) according to the manufacturer’s instructions. Sample dilutions were prepared 1:2 for CCL2, CX3CR1, GM-CSF and TNFα, and 1:10 for E-selectin, MAD-CAM and M-CSF.

### Quantitative real time PCR (qRT-PCR)

Total RNA from the ventral subregion of the hippocampus was isolated by single step guanidinium isothiocyanate/phenol extraction using PureZol RNA isolation reagent (Bio-Rad Laboratories, Italy) according to the manufacturer’s instructions. The samples were processed for real-time reverse transcription polymerase chain reaction (real time RT-PCR) to assess mRNA levels, as previously described [70]. The complete list of primers and probes with the relative sequences are listed in the supplementary materials.

### Western blot

Proteins from the nuclear and cytosolic fractions of hippocampi were isolated and quantified according to the Bradford Protein Assay procedure (Bio-Rad, Milan, Italy), as previously described [71]. Results were standardized to β-actin control protein detected at 43 kDa. As gels were run in duplicate, the results from the two gels were averaged with a correction factor: correction factor gel B = average of (OD protein of interest/OD β-actin for each sample loaded in gel A)/(OD protein of interest/OD β-actin for the same sample loaded in gel B) [61]. All image analysis were conducted in a blinded manner. The full list of antibodies, ECL reagents (Cyanagen, Italy) and the full cropped immunoblots are reported in the Supplementary Figs. 2–5.

### Statistical analysis

Data are presented as mean ± SEM. Between-group differences were assessed using unpaired or paired Student’s t tests, or one- or two-way ANOVA as appropriate, followed by Tukey’s post hoc test. To account for potential confounding effects of participant age in the human cohort, we performed a multiple linear regression analysis including age as a covariate for the main outcome parameters, using Prism 11. Age did not emerge as a significant confounding factor, and the full statistical tables are provided in the Supplementary Information (Supplementary Tables 20–21).

Behavioral parameters in rats (body weight, food intake, wheel running) were analyzed by one- or two-way repeated-measures ANOVA. Molecular data were tested for normality and for variance homogeneity. Data were analyzed using ANOVA or parametric or non-parametric Student’s t tests according to the results of normality and variance homogeneity. Correlations were assessed using Pearson’s correlation and linear regression. Outliers (>2 SD) were excluded. Statistical analyses were performed using GraphPad Prism 11, with significance set at p < 0.05; detailed statistics are reported in the Supplementary Tables 3–19.

Further technical details of the materials and methods are reported in the supplementary information.

## Supplementary information

supplementary materials

## Data Availability

The data that support the findings of this study are available from the corresponding author upon reasonable request.
