# Supplementary material for: Glucocorticoid signalling dysregulation and immune cell adaptation in Anorexia Nervosa
Source: Mol Psychiatry. 2026 Jul 20;31(12):6984–98. doi: 10.1038/s41380-026-03756-4 (PMC13736517; doi:10.1038/s41380-026-03756-4)
Supplement: Supplementary file 1 [file 41380_2026_3756_MOESM1_ESM.docx]

**Supplementary Information**

**Protein isolation and western blot**

Proteins from hippocampi were homogenized using a cold buffer containing 0,32M sucrose, 0,1mM PMSF, 1mM HEPES, 0,1mM EGTA pH 7,4, in presence of commercial cocktails of protease (Roche) and phosphatase (Sigma-Aldrich) inhibitors. Total amount of proteins in both fractions was quantified according to the Bradford Protein Assay procedure (Bio-Rad, Milan, Italy, with bovine serum albumin as calibration standard. Eight micrograms of proteins for each sample were run on a sodium dodecyl sulfate-8% polyacrylamide gel under reducing conditions and electrophoretically transferred onto nitrocellulose membrane (Bio-Rad Laboratories). Blots were blocked 1 h at room temperature with I-Block solution (Life Technologies Italia) in TBS + 0.1% Tween-20 buffer or BSA5% in TBS buffer, incubated with antibodies against the proteins of interest. Immunocomplexes were visualized by chemiluminescence (WESTAR ECL reagents, Cyanagen Srl) using the Chemidoc MP Imaging System (Bio-Rad Laboratories) and analyzed with Image LabTM software (Bio-Rad) by evaluating the band density.

**Quantitative real time PCR (qRT-PCR)**

After RNA isolation isolation, each sample aliquot was treated with DNase I (RNase-free buffer with MnCl₂; Thermo Scientific) at 37°C for 30 min to remove residual genomic DNA, followed by incubation at 65°C for 10 min with EDTA to inactivate the enzyme. Gene expression was assessed by TaqMan qRT-PCR using the iScript™ One-Step RT-PCR Kit for probes (Bio-Rad) on a CFX384 Real-Time System (Bio-Rad). Reactions were run in triplicate in 384-well plates under the following conditions: 50°C for 10 min (reverse transcription), 95°C for 5 min (enzyme activation), and 39 cycles of 95°C for 10 s and 60°C for 30 s. Relative expression levels were calculated by the ΔΔCt method using 36B4 as the internal reference gene.

**Detailed description of ABA procedure**

**Experiment 1**

Rats were group-housed after their arrival at post-natal day (P) 28, left undisturbed in their cages to get accustomed to the room and the dark/light cycle for 7 days and fed with food and water *ad libitum*. The ABA protocol was set up according to our literature^35–37^. In brief, animals were individually housed (P35) in different cages and randomly subdivided into four experimental groups: [1] control (CTRL) group: sedentary + food *ad libitum*; [2] food-restricted (FR) group: sedentary + food restriction (food access limited for 2h/day); [3] exercise (EXE) group: voluntary running activity in a mechanical wheel + food *ad libitum*; [4] activity-based anorexia (ABA) group: voluntary running activity in a mechanical wheel + food restriction (food access limited for 2h/day). At the beginning of the experimental paradigm (Fig. 3a), all animals started the habituation period (P35-P38): CTRL and FR groups were kept in classical home cages, EXE and ABA groups were placed in dedicated activity cages equipped with a mechanical activity wheel (activity wheel BIO-ACTIVW-R cage, Bioseb, France). From P38 FR and ABA rodents had access to the food pellets for 2h/day from 10.30 to 12.30 am, period during which the wheels were blocked to preclude rats from privileging run over food.

Body weight, food intake, and running wheel activity were longitudinally monitored in each animal from acclimation (P35) to the end of the experiment, with daily weight measurements, food intake assessed during a 2-h restricted feeding period, and locomotor activity continuously recorded and averaged over 24 h.

Animals were sacrificed at three different timepoints: at PND40, during phenotype induction, at PND42, as they reached the acute phase of the anorexic phenotype, and PND49, after one week of BW recovery as sedentary with food *ad libitum*.

**Supplementary figure 1** Running activity on the wheel in terms of brief exercise accesses counts expressed as the total number of access lower than 2 meters exhibited by ABA rats. Results are presented as the mean ± SEM. Two-way ANOVA with repeated measures followed by Šidák’s multiple comparison test (Time x Treatment: F (6, 108) = 1.743, p=0.1179; Time: F (2.807, 50.52) = 6.174, p=0.0015; Treatment: F (1, 18) = 0.7329, p=0.4032; Subject: F (18, 108) = 6.810, p<0.0001).

**Experiment 2**

At P35 (Fig. 5a), a second cohort of female rats were singly housed and arbitrarily divided into [1] CTRL-SAL: sedentary + food *ad libitum* + saline; [2] CTRL-RU486: sedentary + food *ad libitum* + RU486 10mg/kg; ABA): [3] ABA-SAL: voluntary running activity in a mechanical wheel + food restriction (food access limited for 2h/day) + saline; [4] ABA-RU486: voluntary running activity in a mechanical wheel + food restriction (food access limited for 2h/day) + RU486 10mg/kg.

The pharmacological treatment started at P40: ABA-SAL and ABA-RU486 were submitted to 2 intraperitoneal injections of saline or RU486 (10mg/kg) per day (9:00am-9:00pm). At P42 all the animals were sacrificed. After decapitation, the hippocampi were grossly dissected from 2 mm slice following the coordinates (-1.72 mm to -6.84) of the Rat Brain Atlas of Paxinos and Watson^66^, frozen on dry ice, and stored at -80°C. Trunk blood from each rat was collected immediately after decapitation as well.

Body weight and food intake were evaluated per each animal from the first day of acclimatation (P35) until the end of the experiment (P40, P42 or P49). Rats were weighted daily between 9.00 and 9.30 am, before the dark shift. Food intake was measured with a specific milligram-sensitive scale before the beginning of the 2h of food restriction and immediately after and calculated as grams of food given at the beginning of the 2h of food access – grams weighted after the 2 hours.

Running wheel activity was assessed by means of specific activity cages equipped with mechanical wheels (Activity wheel BIO-ACTIVW-R cage, Bioseb, Vitrolles, France) connected to a counter device associated with a monitoring software (BIO-ACTIVW-SOFT v1.2.1, Bioseb, Vitrolles, France). Running wheel data were recorded at 30 minutes intervals throughout the whole experiment and the distance travelled (m) is presented as average for each animal during the 24h of recording. The habituation period was scheduled to reduce potential different individual aptitudes to exercise activity, to set the running baseline and to accustom animals to the interaction with the investigator.

**Detailed statistical analysis**

Data were collected as independent determinations and are presented as means ± standard error of the mean (SEM). Between groups differences in clinical parameters were assessed using Unpaired Student’s t test or one-way analysis of variance (ANOVA). Pre-post (T0-T1) analysis of clinical parameters were performed on the same patients tested at the first clinical evaluation (T0) and after one year (T1) and the Paired Student’s t test was used.

Behavioral data related to the characterization of the anorexic phenotype in rats, such as body weight, food intake, and distance travelled on the wheel were assessed by one-way (Experiment 2) or two-way (Experiment 1) ANOVA with repeated measures, followed by Tukey’s multiple comparisons test.

Molecular changes in mRNA and protein levels were tested for normality of residuals with the Kolmogorov-Smirnov test. Data from Experiment 1 with normal distribution were analyzed by two-way ANOVA, using as control condition the combination of food *ad libitum* and no physical exercise (CTRL), and as the testing condition the combination of food restriction and physical exercise (ABA), which were considered as independent variables at the three time points analyzed: P40, P42 or P49. Data with a non-normal distribution were analyzed by the Mann Whitney test (U). Gene and protein expression data from Experiment 2 with normal distribution were analyzed by one-way ANOVA to assess differences among the three independent groups: CTRL-SAL, ABA-SAL and ABA-RU486; corticosterone levels from Experiment 2 were analyzed by two-way ANOVA (independent variables: treatment and ABA procedure). Both the ANOVA analyses were followed by Tukey's multiple comparison post hoc test.

Pearson’s product-moment coefficients (r) and linear regression analyses (R^2^) were calculated to study potential correlations between behavioral outcomes induced by the ABA procedure and corticosterone levels of ABA rodents.

Subjects were eliminated from the final dataset if their data deviated from the mean by 2 SDs. Prism 11 (GraphPad Software Prism v11, San Diego, CA, USA) was used for the analysis of all data. Significance for all tests was assumed at p<0,05. Detailed statistics is reported in the supplementary materials in Tables 3-19.

**Biochemical results**

|  | **HC** (n=26)  *(± SD)* | **AN T0** (n=35) *(± SD)* | **AN T1** (n=20)  *(± SD)* | **AN T0 vs HC**  p value | **AN T1 vs HC**  p value | **AN T0 vs AN T1**  p value |
| --- | --- | --- | --- | --- | --- | --- |
| **Glucose**  74 - 106 mg/dl | 82,85 (6,31) | 82,20 (4,76) | 80,30 (3,16) | 0,0270 * | 0,8615 | 0,0375 * |
| **Uric Acid**  7 - 17 mg/dl | 4,30 (0,73) | 12,60 (8,91) | 14,90 (4,01) | <0,0001 *** | <0,0001 *** | 0,9190 |
| **Creatinine**  0.52 - 1.04 mg/dl | 0,75 (0,09) | 0,73 (0,15) | 0,73 (0,10) | 0,2227 | 0,3680 | 0,6152 |
| **Alanine Aminotransferase (ALT)**  < 35 U/l | 15,96 (7,14) | 58,21 (83,14) | 27,27 (20,67) | <0,0001*** | 0,0008*** | 0,5345 |
| **Aspartate Aminotransferase (AST)**  14 - 36 U/l | 18,85 (4,28) | 40,21 (18,93) | 30,82 (4,56) | <0,0001 *** | <0,0001 *** | 0,5932 |
| **Albumin**  3.5 - 5 g/dl | 4,39 (0,31) | 4,73 (0,30) | 8,13 (15,51) | 0,0004 *** | 0,0028 ** | 0,3801 |
| **Lactate Dehydrogenase (LDH)**  120 - 246 U/l | 162,74 (20,34) | 182,88 (33,15) | 187,95(46,71) | 0,0110 * | 0,0300 * | 0,8611 |
| **Alkaline Phosphatase** **(ALP)**  38 - 126 U/l | 60,74 (12,97) | 60,67 (17,77) | 65,92(14,88) | 0,2836 | 0,1197 | 0,0072 |
| **Gamma-Glutamyl Transferase**  12 - 58 U/l | 15,88 (8,97) | 20,00 (10,99) | 15,55 (6,89) | 0,3531 | 0,8559 | 0,3891 |

**Table 3** Biochemical parameters obtained from the blood tests of patients with AN and HC at both T0 and T1. The ranges shown beneath the parameters are for reference only and reflect the physiological intervals typically adopted in clinical settings. Results are presented as raw data ± SD. Unpaired Student’s t test. Statistical significance is calculated at p<0.05 (*); (**p<0.01, ***p<0.001). Detailed statistics is presented in supplementary table 4.

**Supplementary table 1.** List of antibodies used for FACS and details of dilution.

| **Protein of interest** | **Antibody diluition** |
| --- | --- |
| **Granulocytes RP-1** (BD Bioscience, RRID AB_2741250) | 1:1000 |
| **CD4** (BD Bioscience, RRID AB_2871125) | 1:1000 |
| **CD45R** (BD Bioscience, RRID AB_2872863) | 1:1000 |
| **CD45** (BD Bioscience, RRID AB_2740403) | 1:1000 |
| **Erythroid APC** (BD Bioscience, RRID AB_11152955) | 1:1000 |
| **CD8a** (BD Bioscience, RRID AB_2740001) | 1:1000 |
| **CD3 (**BD Bioscience, RRID AB_2738503) | 1:1000 |
| **CD62L** (BD Bioscience, RRID AB_2743343) | 1:1000 |
| **CD11b** (BD Bioscience, RRID AB_395627) | 1:1000 |

**List of primer and probe sequences for quantitative real time PCR (qRT-PCR)**

RT-PCR analyses were performed to evaluate *total* *Tnf-a, Trem2, Iba1, Cd11b, CD68, Cx3cr1, Grα, Gilz, Sgk1* gene expression. Primers and probe for *Tnf-a, Trem2, Iba1, Cd11b, CD68, Cx3cr1* were purchased from Applied Biosystem, Foster City, California (Tnf-a (Rn99999017_m1), Trem2 (Rn01512170_m1), Iba1 (Rn03993468_g1), Cd11b (Rn00709342_m1), CD68 (Rn01495634_g1), Cx3cr1 (Rn02134446_s1). Primers and probe for *Grα, Grβ, Gilz, Fkbp5, , Sgk1* and *36B4* were purchased from Eurofins MWG-Operon. Their sequences are shown: *Grα:* forward primer 5’-GCGACAGAAGCAGTTGAGTCATC-3’, reverse primer 5′-CCATGCCTCCACGTAACTGTTAG-3’, probe 5’-TGAAGTGATAGCACAGCAGACAGTGA-3′; *Grβ:* forward primer 5’- GCGCTTGAGGCTAAGATAGCT-3’, reverse primer 5′- CCCATGTTTCTGCCTCTTTCTTTG-3’, probe 5’- AGTCTGCCTTCAGAATGCCTGTCA-3′; *Gilz:* forward primer 5’-CGGTCTATCAACTGCACAATTTC-3’, reverse primer 5’-CTTCACTAGATCCATGGCCTG-3’, probe 5’-AACGGAAACCACATCCCCTCCAA-3’; *Fkbp5:* forward primer 5’- GAACCCAATGCTGAGCTTATG-3’, reverse primer 5’- ATGTACTTGCCTCCCTTGAAG-3’, probe 5’- TGTCCATCTCCCAGGATTCTTTGGC-3’; *Sgk1* forward primer 5’-GACTACATTAATGGCGGAGAGC-3’, reverse primer 5′-AGGGAGTGCAGATAACCCAAG-3’, probe 5’-TGCTCGCTTCTACGCAGC-3’; *36B4*: forward primer 5′-TTCCCACTGGCTGAAAAGGT-3′, reverse primer 5′-CGCAGCCGCAAATGC-3′, probe 5′-AAGGCCTTCCTGGCC GATCCATC-3′.

**Supplementary table 2.** List of antibodies used for western blot and details of the incubation conditions.

| **Protein of interest** | **Primary antibody** | **Secondary antibody** |
| --- | --- | --- |
| **pGR (95kDa)**  (abcam, RRID: AB_941772) | 1:1000 BSA  o/n 4°C | 1:1000 anti-Rabbit IgG BSA  1h RT |
| **GR (95kDa)**  (Invitrogen, RRID: AB_2283110) | 1:1000 BSA  o/n 4°C | 1:1000 anti-Rabbit IgG BSA  1h RT |
| **FKBP5 (52kDa)**  (abcam, RRID: AB_11128515) | 1:1000 iBlock  o/n 4°C | 1:1000 anti-Rabbit IgG iBlock  1h RT |
| **b-actin (43KkDa)**  (SIGMA, RRID: AB_476743) | 1:7500 iBlock  1h RT | 1:10000 anti-Mouse IgG iBlock  1hRT |

**Supplementary table 3.** Detailed statistical values of the data presented in table 1 relative to clinical and anthropometric characteristics, EDIs and SCL90 evaluation.

| **Unpaired Student’s t test**  **(two tailed)**  **ANt0 vs ANt1** | **Mean difference ± SEM** | **t, dF** | **p value** |
| --- | --- | --- | --- |
| **Bulimia** | -0,4125 ± 1,812 | t=0,2277, df=19 | 0,8223 |
| **Body dissatisfaction** | 4,550 ± 3,686 | t=1,235, df=23 | 0,2295 |
| **Mann-Whitney test**  **ANt0 vs ANt1** | **Median differences** | **Mann-Whitney U** | **p value** |
| **Drive for thinnes** | -3,000 | 38,50 | 0,5469 |
| **SCL90 global score** | -0,07500 | 42 | 0,6210 |

**Supplementary table 4.** Detailed statistical values of the Unpaired Student’s or Mann-Whitney test performed on blood test counts presented in table 2 and 3.

| **Unpaired Student’s t test**  **(two tailed)**  **HC vs ANt0** | **Mean difference ± SEM** | **t, dF** | **p value** |
| --- | --- | --- | --- |
| **Red Blood Cells (RBC)** | -0,3212 ± 0,09648 | t=3,329, df=59 | 0,0015 |
| **Hemoglobin** | -0,3359 ± 0,2297 | t=1,462, df=39,57 | 0,1515 |
| **Hematocrit** | -0,8740 ± 0,5816 | t=1,503, df=59 | 0,1382 |
| **Mean Corpuscular Volume (MCV)** | 4,543 ± 1,132 | t=4,012, df=59 | 0,0002 |
| **Mean Corpuscular Hemoglobin** **(MCH)** | 3,812 ± 0,3941 | t=9,671, df=36,02 | <0,0001 |
| **Platelets** | -49,62 ± 15,51 | t=3,199, df=59 | 0,0022 |
| **Glucose** | -4,446 ± 1,960 | t=2,268, df=59 | 0,0270 |
| **Alkaline Phosphatase** **(ALP)** | -4,296 ± 3,964 | t=1,084, df=51 | 0,2836 |
| **Mann-Whitney test**  **HC vs ANt0** | **Median differences** | **Mann-Whitney U** | **p value** |
| **Mean Corpuscular Hemoglobin Concentration** **(MCHC)** | -2,300 | 111 | <0,0001 |
| **Basophil %** | 0,2000 | 368 | 0,2050 |
| **Basophils (Absolute Count)** | 0,000 | 356 | 0,1409 |
| **Eosinophil %** | 0,000 | 434 | 0,7636 |
| **Eosinophil (Absolute Count)** | 0,01000 | 411 | 0,5161 |
| **Uric Acid** | 9,700 | 21,50 | <0,0001 |
| **Creatinine** | -0,03500 | 371,5 | 0,2227 |
| **Alanine Aminotransferase (ALT)** | 8,500 | 195 | <0,0001 |
| **Aspartate Aminotransferase (AST)** | 15,00 | 47,50 | <0,0001 |
| **Albumin** | 0,3500 | 143 | 0,0004 |
| **Lactate Dehydrogenase (LDH)** | 26,00 | 193,5 | 0,0110 |
| **Gamma-Glutamyl Transferase** | 2,000 | 391 | 0,3531 |
| **Unpaired Student’s t test**  **(two tailed)**  **HC vs ANt1** | **Mean difference ± SEM** | **t, dF** | **p value** |
| **Red Blood Cells (RBC)** | -0,2578 ± 0,1217 | t=2,119, df=44 | 0,0397 |
| **Hemoglobin** | -0,3731 ± 0,2848 | t=1,310, df=44 | 0,1970 |
| **Hematocrit** | -0,5904 ± 0,7516 | t=0,7855, df=44 | 0,4364 |
| **Mean Corpuscular Volume (MCV)** | 3,995 ± 1,444 | t=2,766, df=44 | 0,0083 |
| **Mean Corpuscular Hemoglobin Concentration** **(MCHC)** | -2,742 ± 0,5467 | t=5,016, df=24,24 | <0,0001 |
| **Mean Corpuscular Hemoglobin** **(MCH)** | 3,218 ± 0,4219 | t=7,628, df=40,48 | <0,0001 |
| **Platelets** | -24,88 ± 20,23 | t=1,230, df=44 | 0,2252 |
| **Basophil %** | 0,08308 ± 0,1012 | t=0,8213, df=44 | 0,4159 |
| **Glucose** | 0,3538 ± 2,017 | t=0,1755, df=44 | 0,8615 |
| **Aspartate Aminotransferase (AST)** | 12,40 ± 1,263 | t=9,823, df=44 | <0,0001 |
| **Alkaline Phosphatase** **(ALP)** | 6,963 ± 4,371 | t=1,593, df=37 | 0,1197 |
| **Mann-Whitney test**  **HC vs ANt1** | **Median differences** | **Mann-Whitney U** | **p value** |
| **Basophils (Absolute Count)** | 0,000 | 247,5 | 0,7840 |
| **Eosinophil %** | 0,6500 | 204,5 | 0,2226 |
| **Eosinophil (Absolute Count)** | 0,01000 | 201,5 | 0,1918 |
| **Uric Acid** | 9,200 | 0 | <0,0001 |
| **Creatinine** | -0,03500 | 219 | 0,3680 |
| **Alanine Aminotransferase (ALT)** | 7,500 | 112,5 | 0,0008 |
| **Albumin** | 0,1500 | 86 | 0,0028 |
| **Lactate Dehydrogenase (LDH)** | 21,00 | 106,5 | 0,0300 |
| **Gamma-Glutamyl Transferase** | 1,000 | 251,5 | 0,8559 |
| **Unpaired Student’s t test**  **(two tailed)**  **ANt0 vs ANt1** | **Mean difference ± SEM** | **t, dF** | **p value** |
| **Red Blood Cells (RBC)** | 0,06336 ± 0,1121 | t=0,5653, df=53 | 0,5743 |
| **Hemoglobin** | -0,03714 ± 0,2051 | t=0,1811, df=53 | 0,8570 |
| **Hematocrit** | 0,2836 ± 0,6330 | t=0,4480, df=53 | 0,6560 |
| **Mean Corpuscular Volume (MCV)** | -0,5486 ± 1,401 | t=0,3915, df=53 | 0,6970 |
| **Mean Corpuscular Hemoglobin Concentration** **(MCHC)** | -0,2364 ± 0,6032 | t=0,3920, df=53 | 0,6967 |
| **Basophil %** | 0,01286 ± 0,08084 | t=0,1591, df=53 | 0,8742 |
| **Glucose** | 4,800 ± 2,250 | t=2,134, df=53 | 0,0375 |
| **Uric Acid** | 0,1286 ± 1,258 | t=0,1022, df=53 | 0,9190 |
| **Alkaline Phosphatase** **(ALP)** | 11,26 ± 4,022 | t=2,799, df=52 | 0,0072 |
| **Mann-Whitney test**  **ANt0 vs ANt1** | **Median differences** | **Mann-Whitney U** | **p value** |
| **Mean Corpuscular Hemoglobin** **(MCH)** | -0,6000 | 238,5 | 0,0507 |
| **Platelets** | 18,50 | 265 | 0,1390 |
| **Basophils (Absolute Count)** | 0,000 | 283 | 0,2344 |
| **Eosinophil %** | 0,6500 | 263 | 0,1294 |
| **Eosinophil (Absolute Count)** | 0,000 | 249 | 0,0575 |
| **Creatinine** | 0,000 | 321,5 | 0,6152 |
| **Alanine Aminotransferase (ALT)** | -1,000 | 314 | 0,5345 |
| **Aspartate Aminotransferase (AST)** | -2,000 | 319 | 0,5932 |
| **Albumin** | -0,2000 | 299,5 | 0,3801 |
| **Lactate Dehydrogenase (LDH)** | -5,000 | 322,5 | 0,8611 |
| **Gamma-Glutamyl Transferase** | -1,000 | 300,5 | 0,3891 |

**Supplementary table 5.** Detailed statistical values of the Unpaired Student’s or Mann-Whitney test performed on data presented in Figure 1, panels b, c, f, l-v, one sample t test showed in Figure 1, panel d, and Pearson’s product–moment correlation (r) analyses and linear regression analyses (R^2^) presented in Figure 1 panels h-k.

| **Unpaired Student’s t test**  **(two tailed)**  **HC vs AN_t0_** | **Mean difference ± SEM** | **t, dF** | **p value** |
| --- | --- | --- | --- |
| **Body weight** | -12,90 ± 1,709 | t=7,546, df=62 | <0.0001 |
| **BMI** | -4,962 ± 0,5775 | t=8,593, df=63 | <0.0001 |
| **EED19** | -0,8006 ± 0,1868 | t=4,287, df=53 | <0.0001 |
| **Cortisol** | -42,06 ± 18,79 | t=2,238, df=59 | 0,0290 |
| **Circulating HSCs** | 6,260 ± 2,762 | t=2,266, df=18 | 0,0360 |
| **Leukocytes** | 7113 ± 2106 | t=3,377, df=14 | 0,0045 |
| **Neutrophils** | 3255 ± 1443 | t=2,255, df=15 | 0,0395 |
| **Monocytes** | 589,2 ± 209,7 | t=2,810, df=14 | 0,0139 |
| **Lymphocytes** | 2405 ± 1017 | t=2,365, df=14 | 0,0330 |
| **CD62L MFI Leukocytes** | -0,4120 ± 0,1647 | t=2,502, df=15 | 0,0244 |
| **CD62L MFI Neutrophils** | -0,3580 ± 0,1596 | t=2,243, df=14 | 0,0416 |
| **CD62L MFI Monocytes** | -0,5072 ± 0,1412 | t=3,593, df=15 | 0,0027 |
| **CD62L MFI Lymphocytes** | -0,3294 ± 0,1384 | t=2,381, df=15 | 0,0310 |
| **CX3CL1** | -0,06747 ± 0,06258 | t=1,078, df=58 | 0,2855 |
| **GM-CSF** | 0,2748 ± 0,1355 | t=2,028, df=59 | 0,0471 |
| **E-Selectin** | 0,07086 ± 0,1260 | t=0,5622, df=59 | 0,5761 |
| **MAD-CAM** | 0,06429 ± 0,05169 | t=1,244, df=59 | 0,2185 |
| **Mann-Whitney test**  **HC vs ANt0** | **Median differences** | **Mann-Whitney U** | **p value** |
| **CCL2** | 0,02000 | 432 | 0,7659 |
| **TNFα** | -0,06000 | 418,5 | 0,5993 |
| **M-CSF** | -0,1600 | 213,5 | 0,0003 |
| **One sample t and Wilcoxon** | **μ0** | **Wilcoxon W** | **p value** |
| **AN severity** | **18.5** | **-630.0** | <0,0001 |
| **Correlations** | **Pearson r** | **R^2^** | **p value** |
| **Compulsive exercise - HC** | 0,3665 | 0,1343 | 0,0854 |
| **Compulsive exercise – AN_t0_** | 0,9009 | 0,8117 | <0,0001 |
| **Awareness of bodily signal - HC** | 0,4530 | 0,2052 | 0,0230 |
| **Awareness of bodily signal – AN_t0_** | 0,4708 | 0,2216 | 0,0075 |
| **Exercise for weight and shape reasons - HC** | 0,3215 | 0,1034 | 0,1171 |
| **Exercise for weight and shape reasons - An_t0_** | 0,6204 | 0,3849 | 0,0002 |
| **Positive healthy exercise - HC** | 0,4374 | 0,1913 | 0,0288 |
| **Positive healthy exercise - AN_t0_** | 0,2190 | 0,04796 | 0,2366 |

**Supplementary table 6.** Detailed statistical values of the Paired Student’s t test, Unpaired Student’s T test, One-way ANOVA and Mann-Whitney test performed on data presented in Figure 2 panels b-i, k, l.

| **Paired Student’s t test (two tailed)**  **ANt0 vs ANt1** | **Mean difference ± SEM** | **t, dF** | **p value** |
| --- | --- | --- | --- |
| **Body weight** | -7,405 ± 1,953 | t=3,792, df=19 | 0,0012 |
| **BMI** | -2,606 ± 0,6813 | t=3,825, df=19 | 0,0011 |
| **Cortisol** | -52,76 ± 17,60 | t=2,998, df=19 | 0,0074 |
| **Leukocytes** | -0,6586 ± 0,2756 | t=2,389, df=20 | 0,0268 |
| **Neutrophils** | -0,4486 ± 0,2799 | t=1,603, df=20 | 0,1247 |
| **Monocytes** | -0,07857 ± 0,02638 | t=2,979, df=20 | 0,0074 |
| **Lymphocytes** | -0,08952 ± 0,1045 | t=0,8566, df=20 | 0,4018 |
| **Unpaired Student’s t test (two tailed)**  **ANt0 vs ANt1** | **Mean difference ± SEM** | **t, dF** | **p value** |
| **Body weight** | -7,405 ± 2,386 | t=3,104, df=38 | 0,0036 |
| **BMI** | -2,606 ± 0,7170 | t=3,634, df=38 | 0,0008 |
| **Cortisol** | -52,76 ± 25,57 | t=2,063, df=38 | 0,0460 |
| **Leukocytes** | -0,6586 ± 0,5669 | t=1,162, df=40 | 0,2522 |
| **Neutrophils** | -0,4486 ± 0,4136 | t=1,085, df=40 | 0,2846 |
| **Monocytes** | -0,07857 ± 0,05190 | t=1,514, df=40 | 0,1379 |
| **Lymphocytes** | -0,08485 ± 0,1978 | t=0,4289, df=41 | 0,6702 |
| **CX3CL1** | 0,03464 ± 0,07153 | t=0,4843, df=50 | 0,6303 |
| **GM-CSF** | -0,2048 ± 0,1884 | t=1,087, df=50 | 0,2822 |
| **TNFα** | -0,2271 ± 0,07504 | t=3,026, df=48 | 0,0040 |
| **MAD-CAM** | -0,1167 ± 0,06943 | t=1,681, df=50 | 0,0990 |
| **M-CSF** | -0,1252 ± 0,08233 | t=1,521, df=50 | 0,1347 |
| **Mann-Whitney test**  **ANt0 vs ANt1** | **Median differences** | **Mann-Whitney U** | **p value** |
| **CCL2** | -0,02612 | 267 | 0,7979 |
| **E-Selectin** | 0,4458 | 147 | 0,0028 |
| **One-way ANOVA**  **(HC, ANt1 rec, ANt1 non rec)** | **F (DFn, DFd)** | **p value** |  |
| **Cortisol** | F (1,918, 41,24) = 15,06 | <0,0001 |  |
| **Mixed-effects analysis** | **Group** | **Mean differences** | **p value** |
| **Cortisol** | **HC vs AN_t1 recovered_** | -94,60 | 0,0165 |
|  | **HC vs AN_t1_** _non recovered_ | -105,7 | 0,0194 |
| **Unpaired Student’s t test (two tailed)**  **Non recovered vs Recovered** | **Mean difference ± SEM** | **t, dF** | **p value** |
| **CX3CL1** | 0,1032 ± 0,1236 | t=0,8346, df=14 | 0,4180 |
| **GM-CSF** | -0,1846 ± 0,3309 | t=0,5580, df=13 | 0,5863 |
| **E-Selectin** | 0,6004 ± 0,3365 | t=1,784, df=12 | 0,0996 |
| **MAD-CAM** | -0,02032 ± 0,1217 | t=0,1669, df=14 | 0,8698 |
| **M-CSF** | 0,1310 ± 0,1652 | t=2,219, df=13 | 0,0449 |
| **Mann-Whitney test**  **Non recovered vs Recovered** | **Median differences** | **Mann-Whitney U** | **p value** |
| **CCL2** | 0,06000 | 29 | 0,8371 |
| **TNFα** | 0,2300 | 10,50 | 0,0247 |
| **Unpaired Student’s t test (two tailed)**  **Non recovered vs Ant0** | **Mean difference ± SEM** | **t, dF** | **p value** |
| **CX3CL1** | 0,09429 ± 0,09921 | t=0,9503, df=40 | 0,3477 |
| **GM-CSF** | -0,1671 ± 0,2504 | t=0,6674, df=40 | 0,5083 |
| **E-Selectin** | -0,3320 ± 0,1719 | t=1,931, df=38 | 0,0610 |
| **MAD-CAM** | -0,1314 ± 0,09978 | t=1,317, df=40 | 0,1953 |
| **M-CSF** | 0,1033 ± 0,1128 | t=0,9162, df=39 | 0,3652 |
| **Mann-Whitney test**  **Non recovered vs Ant0** | **Median differences** | **Mann-Whitney U** | **p value** |
| **CCL2** | 0,06413 | 113 | 0,8459 |
| **TNFα** | -0,04965 | 97 | 0,6033 |
| **Unpaired Student’s t test (two tailed) Recovered vs Ant0** | **Mean difference ± SEM** | **t, dF** | **p value** |
| **CX3CL1** | -0,008889 ± 0,09353 | t=0,09504, df=42 | 0,9247 |
| **GM-CSF** | 0,01750 ± 0,2171 | t=0,08060, df=41 | 0,9362 |
| **TNFα** | -0,2900 ± 0,07940 | t=3,652, df=39 | 0,0008 |
| **E-Selectin** | -0,9324 ± 0,1918 | t=4,862, df=40 | <0,0001 |
| **MAD-CAM** | -0,1111 ± 0,08847 | t=1,256, df=42 | 0,2161 |
| **M-CSF** | -0,1667 ± 0,09202 | t=1,811, df=42 | 0,0773 |
| **Mann-Whitney test**  **Recovered vs Ant0** | **Median differences** | **Mann-Whitney U** | **p value** |
| **CCL2** | 0,004127 | 132 | 0,5420 |

**Supplementary table 7.** Detailed statistical values of the main effects of the Two-way ANOVA performed on behavioral measurements presented in Figure 3, panels b-f.

| **Two-way ANOVA** |  | ***F (DFn, DFd)*** | ***P value*** |
| --- | --- | --- | --- |
| **Body weight** | Time | F (19, 840) = 1396 | <0,0001 |
|  | Group | F (3, 55) = 40.00 | <0,0001 |
|  | Time x group | F (57, 840) = 89.30 | <0,0001 |
| **Food intake** | Time | F (13, 500) = 93,93 | <0.0001 |
|  | Group | F (3, 55) = 35,95 | <0.0001 |
|  | Time x group | F (39, 500) = 42,19 | <0.0001 |
| **Distance increase** | Time | F (6, 160) = 40,69 | <0.0001 |
|  | Group | F (1, 27) = 23,14 | 0,0001 |
|  | Time x group | F (6, 160) = 7,845 | <0.0001 |
| **Mean speed** | Time | F (6, 162) = 5,230 | <0.0001 |
|  | Group | F (6, 162) = 24,07 | 0,2059 |
|  | Time x group | F (1, 27) = 10,68 | <0.0001 |
| **Long sequence count** | Time | F (6, 162) = 11,87 | <0.0001 |
|  | Group | F (6, 162) = 25,84 | 0,0030 |
|  | Time x group | F (1, 27) = 8,931 | <0.0001 |

**Supplementary table 8.** Detailed statistical analysis of the Tuckey’s or Šidák’s multiple comparison test performed on behavioral measurements presented in Figure 3, panels b-f.

|  |  | **Tuckey’s multiple comparison test** | |
| --- | --- | --- | --- |
| **Body weight** | | *Mean difference (g)* | *Adjusted P value* |
| **PND36** | CTRL vs ABA | 8,870 | 0,0120 |
| **PND37** | CTRL vs EXE | 12,10 | 0,0030 |
|  | CTRL vs ABA | 12,95 | <0,0001 |
| **PND38** | CTRL vs EXE | 12,77 | 0,0014 |
|  | CTRL vs ABA | 12,24 | 0,0001 |
| **PND39** | CTRL vs FR | 14,94 | 0,0001 |
|  | CTRL vs EXE | 15,02 | <0,0001 |
|  | CTRL vs ABA | 28,39 | <0,0001 |
|  | FR vs ABA | 13,45 | 0,0008 |
|  | EXE vs ABA | 13,36 | 0,0008 |
| **PND40** | CTRL vs FR | 28,62 | <0,0001 |
|  | CTRL vs EXE | 15,04 | <0,0001 |
|  | CTRL vs ABA | 45,22 | <0,0001 |
|  | FR vs EXE | -13,59 | 0,0042 |
|  | FR vs ABA | 16,60 | <0,0001 |
|  | EXE vs ABA | 30,18 | <0,0001 |
| **PND41** | CTRL vs FR | 38,64 | <0,0001 |
|  | CTRL vs EXE | 17,26 | <0,0001 |
|  | CTRL vs ABA | 62,33 | <0,0001 |
|  | FR vs EXE | -21,38 | <0,0001 |
|  | FR vs ABA | 23,69 | <0,0001 |
|  | EXE vs ABA | 45,07 | <0,0001 |
| **PND42** | CTRL vs FR | 48,19 | <0,0001 |
|  | CTRL vs EXE | 16,71 | <0,0001 |
|  | CTRL vs ABA | 71,94 | <0,0001 |
|  | FR vs EXE | -31,47 | <0,0001 |
|  | FR vs ABA | 23,76 | <0,0001 |
|  | EXE vs ABA | 55,23 | <0,0001 |
| **PND43** | CTRL vs FR | 43,14 | <0,0001 |
|  | CTRL vs EXE | 16,96 | <0,0001 |
|  | CTRL vs ABA | 55,53 | <0,0001 |
|  | FR vs EXE | -26,18 | <0,0001 |
|  | FR vs ABA | 12,39 | 0,0041 |
|  | EXE vs ABA | 38,57 | <0,0001 |
| **PND44** | CTRL vs FR | 30,99 | <0,0001 |
|  | CTRL vs EXE | 16,65 | 0,0001 |
|  | CTRL vs ABA | 48,17 | <0,0001 |
|  | FR vs EXE | -14,33 | 0,0077 |
|  | FR vs ABA | 17,18 | <0,0001 |
|  | EXE vs ABA | 31,51 | <0,0001 |
| **PND45** | CTRL vs FR | 30,11 | <0,0001 |
|  | CTRL vs EXE | 18,34 | <0,0001 |
|  | CTRL vs ABA | 43,63 | <0,0001 |
|  | FR vs ABA | 13,52 | 0,0034 |
|  | EXE vs ABA | 25,29 | <0,0001 |
| **PND46** | CTRL vs FR | 27,46 | <0,0001 |
|  | CTRL vs EXE | 17,87 | <0,0001 |
|  | CTRL vs ABA | 40,52 | <0,0001 |
|  | FR vs ABA | 13,06 | 0,0051 |
|  | EXE vs ABA | 22,65 | <0,0001 |
| **PND47** | CTRL vs FR | 28,35 | <0,0001 |
|  | CTRL vs EXE | 20,69 | <0,0001 |
|  | CTRL vs ABA | 38,13 | <0,0001 |
|  | EXE vs ABA | 17,44 | <0,0001 |
| **PND48** | CTRL vs FR | 26,90 | <0,0001 |
|  | CTRL vs EXE | 21,17 | <0,0001 |
|  | CTRL vs ABA | 34,93 | <0,0001 |
|  | EXE vs ABA | 13,76 | 0,0027 |
| **PND49** | CTRL vs FR | 27,50 | <0,0001 |
|  | CTRL vs EXE | 22,20 | <0,0001 |
|  | CTRL vs ABA | 30,70 | <0,0001 |
| **Food Intake** |  |  | |
| **PND38** | CTRL vs. ABA | -29,33 | <0,0001 |
|  | FR vs. ABA | -26,13 | 0,0001 |
|  | EXE vs. ABA | -26,96 | <0,0001 |
| **PND39** | CTRL vs. FR | 90,02 | <0,0001 |
|  | CTRL vs. ABA | 71,36 | <0,0001 |
|  | FR vs. EXE | -102,2 | <0,0001 |
|  | FR vs. ABA | -18,65 | <0,0001 |
|  | EXE vs. ABA | 83,56 | <0,0001 |
| **PND40** | CTRL vs. FR | 75,99 | <0,0001 |
|  | CTRL vs. EXE | -20,24 | 0,0045 |
|  | CTRL vs. ABA | 66,67 | <0,0001 |
|  | FR vs. EXE | -96,23 | <0,0001 |
|  | EXE vs. ABA | 86,91 | <0,0001 |
| **PND41** | CTRL vs. FR | 71,27 | <0,0001 |
|  | CTRL vs. EXE | -16,13 | 0,0376 |
|  | CTRL vs. ABA | 61,36 | <0,0001 |
|  | FR vs. EXE | -87,40 | <0,0001 |
|  | EXE vs. ABA | 77,49 | <0,0001 |
| **PND42** | CTRL vs. FR | 68,13 | <0,0001 |
|  | CTRL vs. EXE | -22,63 | 0,0011 |
|  | CTRL vs. ABA | 57,50 | <0,0001 |
|  | FR vs. EXE | -90,76 | <0,0001 |
|  | EXE vs. ABA | 80,12 | <0,0001 |
| **PND43** | FR vs. EXE | -24,94 | 0,0442 |
| **PND44** | CTRL vs. ABA | -32,74 | <0,0001 |
|  | FR vs. EXE | -29,72 | 0,0101 |
|  | FR vs. ABA | -44,21 | <0,0001 |
| **PND45** | CTRL vs. ABA | -39,26 | <0,0001 |
|  | FR vs. ABA | -44,14 | <0,0001 |
|  | EXE vs. ABA | -27,80 | 0,0053 |
| **PND46** | CTRL vs. ABA | -51,42 | <0,0001 |
|  | FR vs. ABA | -47,28 | <0,0001 |
|  | EXE vs. ABA | -34,42 | 0,0003 |
| **PND47** | CTRL vs. ABA | -60,26 | <0,0001 |
|  | FR vs. ABA | -74,42 | <0,0001 |
|  | EXE vs. ABA | -58,74 | <0,0001 |
| **PND48** | CTRL vs. ABA | -52,46 | <0,0001 |
|  | FR vs. ABA | -56,99 | <0,0001 |
|  | EXE vs. ABA | -44,23 | <0,0001 |
| **PND49** | CTRL vs. ABA | -57,51 | <0,0001 |
|  | FR vs. ABA | -61,69 | <0,0001 |
|  | EXE vs. ABA | -53,96 | <0,0001 |
|  |  | **Šidák’s multiple comparison test** | |
| **Distance increase** |  | *Mean difference (g)* | *Adjusted P value* |
| **PND40** | EXE vs ABA | -5191 | <0,0001 |
| **PND41** | EXE vs ABA | -6144 | <0,0001 |
| **PND42** | EXE vs ABA | -4224 | 0,0005 |
| **Mean speed** |  |  | |
| **PND41** | EXE vs ABA | -5,779 | <0,0001 |
| **PND42** | EXE vs ABA | -4,911 | 0,0006 |
| **Sequence count** |  |  | |
| **PND41** | EXE vs ABA | -256,3 | <0,0001 |
| **PND42** | EXE vs ABA | -308,6 | <0,0001 |

**Supplementary table 9.** Detailed statistical values of the main effects of the Two-way ANOVA conducted on data presented in Figure 3, panels g, i-j, related to plasma, blood and bone marrow analysis.

| **PND42** | **Two-way ANOVA** | ***F (DFn, DFd)*** | ***P value*** |
| --- | --- | --- | --- |
| **Corticosterone** | Interaction | F (1, 23) = 1.31 | P=0.2648 |
|  | Exercise | F (1, 23) = 0.0205 | P=0.8873 |
|  | Food restriction | F (1, 23) = 14.6 | P=0.0009 |
| **CD45 cell/ul blood** | Interaction | F (1, 23) = 0,1658 | P=0,6876 |
|  | Exercise | F (1, 23) = 0,6141 | P=0,4413 |
|  | Food restriction | F (1, 23) = 39,85 | P<0,0001 |
| **CD3 cell/ul blood** | Interaction | F (1, 23) = 0,005556 | P=0,9412 |
|  | Exercise | F (1, 23) = 1,073 | P=0,3111 |
|  | Food restriction | F (1, 23) = 15,97 | P=0,0006 |
| **Bcells cell/ul blood** | Interaction | F (1, 23) = 0,005067 | P=0,9439 |
|  | Exercise | F (1, 23) = 1,269 | P=0,2716 |
|  | Food restriction | F (1, 23) = 24,61 | P<0,0001 |
| **Granulocyte cell/ul blood** | Interaction | F (1, 22) = 0,2177 | P=0,6454 |
|  | Exercise | F (1, 22) = 0,1086 | P=0,7449 |
|  | Food restriction | F (1, 22) = 59,36 | P<0,0001 |
| **CD45 cell/ul femur** | Interaction | F (1, 23) = 12,59 | P=0,0017 |
|  | Exercise | F (1, 23) = 0,7393 | P=0,3988 |
|  | Food restriction | F (1, 23) = 0,4569 | P=0,5058 |
| **CD3 % of CD45 femur** | Interaction | F (1, 23) = 0,2218 | P=0,6421 |
|  | Exercise | F (1, 23) = 13,15 | P=0,0014 |
|  | Food restriction | F (1, 23) = 125,2 | P<0,0001 |
| **B cells % of CD45 femur** | Interaction | F (1, 23) = 1,051 | P=0,3159 |
|  | Exercise | F (1, 23) = 18,57 | P=0,0003 |
|  | Food restriction | F (1, 23) = 14,86 | P=0,0008 |
| **Granulocytes % of CD45 femur** | Interaction | F (1, 23) = 0,02073 | P=0,8868 |
|  | Exercise | F (1, 23) = 4,066 | P=0,0556 |
|  | Food restriction | F (1, 23) = 6,880 | P=0,0152 |
| **PND49** | **Two-way ANOVA** | ***F (DFn, DFd)*** | ***P value*** |
| **Corticosterone** | Interaction | F (1, 23) = 4,46 | P=0,0457 |
|  | Exercise | F (1, 23) = 0,760 | P=0,3924 |
|  | Food restriction | F (1, 23) = 4,76 | P=0,0396 |
| **CD45 cell/ul blood** | Interaction | F (1, 21) = 19,27 | P=0,0003 |
|  | Exercise | F (1, 21) = 1,790 | P=0,1953 |
|  | Food restriction | F (1, 21) = 0,2496 | P=0,6226 |
| **CD3 cell/ul blood** | Interaction | F (1, 21) = 18,03 | P=0,0004 |
|  | Exercise | F (1, 21) = 2,677 | P=0,1167 |
|  | Food restriction | F (1, 21) = 0,5543 | P=0,4648 |
| **Bcells cell/ul blood** | Interaction | F (1, 21) = 21,84 | P=0,0001 |
|  | Exercise | F (1, 21) = 3,309 | P=0,0832 |
|  | Food restriction | F (1, 21) = 0,9231 | P=0,3476 |
| **Granulocyte cell/ul blood** | Interaction | F (1, 20) = 5,185 | P=0,0339 |
|  | Exercise | F (1, 20) = 2,487 | P=0,1304 |
|  | Food restriction | F (1, 20) = 1,070 | P=0,3134 |
| **CD45 cell/ul femur** | Interaction | F (1, 21) = 4,168 | P=0,0540 |
|  | Exercise | F (1, 21) = 0,03788 | P=0,8476 |
|  | Food restriction | F (1, 21) = 0,2083 | P=0,6528 |
| **CD3 % of CD45 femur** | Interaction | F (1, 20) = 1,832 | P=0,1910 |
|  | Exercise | F (1, 20) = 0,4152 | P=0,5266 |
|  | Food restriction | F (1, 20) = 1,187 | P=0,2889 |
| **B cells % of CD45 femur** | Interaction | F (1, 21) = 38,70 | P<0,0001 |
|  | Exercise | F (1, 21) = 14,56 | P=0,0010 |
|  | Food restriction | F (1, 21) = 16,70 | P=0,0005 |
| **Granulocytes % of CD45 femur** | Interaction | F (1, 19) = 5,240 | P=0,0337 |
|  | Exercise | F (1, 19) = 6,300 | P=0,0213 |
|  | Food restriction | F (1, 19) = 10,28 | P=0,0046 |

**Supplementary table 10** Detailed statistical analysis of the Two-way ANOVA performed on data presented in Figure 3, panels panels g, i-j related to plasma, blood and bone marrow analysis.

|  |  | **Tuckey’s multiple comparison test** | |
| --- | --- | --- | --- |
| **PND42** | | *Mean difference* | *Adjusted P value* |
| **CD45 cell/ul femur** | CTRL vs EXE | -44,21 | 0,0251 |
|  | EXE vs ABA | 42,37 | 0,0289 |
| **PND49** |  | *Mean difference* | *Adjusted P value* |
| **Corticosterone** | EXE vs ABA | 138 | 0,0300 |
| **CD45 cell/ul blood** | CTRL vs FR | -4163 | 0,0499 |
|  | FR vs ABA | 6129 | 0,0034 |
|  | EXE vs ABA | 5232 | 0,0129 |
| **CD3 cell/ul blood** | FR vs ABA | 2681 | 0,0026 |
|  | EXE vs ABA | 2275 | 0,0110 |
| **Bcells cell/ul blood** | FR vs ABA | 1595 | 0,0010 |
|  | EXE vs ABA | 1384 | 0,0039 |
| **B cells % of CD45 femur** | CTRL vs ABA | 21,02 | <0,0001 |
|  | FR vs ABA | 26,69 | <0,0001 |
|  | EXE vs ABA | 27,41 | <0,0001 |
| **Granulocytes % of CD45 femur** | CTRL vs ABA | -13,34 | 0,0019 |
|  | FR vs ABA | -11,20 | 0,0167 |
|  | EXE vs ABA | -12,83 | 0,0058 |

**Supplementary table 11.** Detailed statistical analysis of the Unpaired Student’s t test performed on data presented in Figure 3, panels q and r related to plasma, blood and bone marrow analysis in ABA rats.

| **Unpaired Student’s t test (two tailed) ABA PND42 vs ABA PND49** | **Mean difference ± SEM** | **t, dF** | **p value** |
| --- | --- | --- | --- |
| **CD45 cell/ul blood** | 654,3 ± 649,5 | t=1,007, df=14 | 0,33 |
| **CD3 cell/ul blood** | -87,20 ± 239,2 | t=0,3645, df=14 | 0,72 |
| **Bcells cell/ul blood** | 112,1 ± 126,3 | t=0,8881, df=14 | 0,39 |
| **Granulocyte cell/ul blood** | 377,6 ± 175,6 | t=2,150, df=13 | 0,05 |
| **CD45 cell/ul femur** | 4,779 ± 14,71 | t=0,3248, df=14 | 0,75 |
| **CD3 % of CD45 femur** | -2,348 ± 0,5360 | t=4,380, df=14 | <0,001 |
| **B cells % of CD45 femur** | -9,690 ± 4,039 | t=2,399, df=14 | 0,03 |
| **Granulocytes % of CD45 femur** | 1,411 ± 5,786 | t=0,2439, df=14 | 0,81 |

**Supplementary table 12.** Detailed statistical values of the main effects of the Two-way ANOVA conducted on data presented in Figure 4, panels a-j, related to protein levels and gene expression analysis.

| **PND40** | **Two-way ANOVA** | ***F (DFn, DFd)*** | ***P value*** |
| --- | --- | --- | --- |
| **GR cytosol** | Interaction | F (1, 19) = 0,8282 | P=0,3742 |
|  | Exercise | F (1, 19) = 2,031 | P=0,1703 |
|  | Food restriction | F (1, 19) = 2,496 | P=0,1307 |
| **GR nucleus** | Interaction | F (1, 20) = 0,08079 | P=0,7792 |
|  | Exercise | F (1, 20) = 0,2883 | P=0,5973 |
|  | Food restriction | F (1, 20) = 0,02494 | P=0,8761 |
| **FKBP5** | Interaction | F (1, 20) = 0,005280 | P=0,9428 |
|  | Exercise | F (1, 20) = 0,2587 | P=0,6166 |
|  | Food restriction | F (1, 20) = 0,2587 | P=0,6166 |
| ***Grα*** | Interaction | F (1, 20) = 7,421 | P=0,0131 |
|  | Exercise | F (1, 20) = 11,16 | P=0,0033 |
|  | Food restriction | F (1, 20) = 14,54 | P=0,0011 |
| ***Grβ*** | Interaction | F (1, 20) = 1,319 | P=0,2644 |
|  | Exercise | F (1, 20) = 8,287 | P=0,0093 |
|  | Food restriction | F (1, 20) = 5,682 | P=0,0272 |
| ***Grα/ Grβ*** | Interaction | F (1, 20) = 0,5071 | P=0,4846 |
|  | Exercise | F (1, 20) = 0,1033 | P=0,7512 |
|  | Food restriction | F (1, 20) = 0,04888 | P=0,8273 |
| ***Gilz*** | Interaction | F (1, 20) = 0,6935 | P=0,4148 |
|  | Exercise | F (1, 20) = 0,5961 | P=0,4491 |
|  | Food restriction | F (1, 20) = 18,53 | P=0,0003 |
| ***Fkbp5*** | Interaction | F (1, 20) = 1,958 | P=0,1770 |
|  | Exercise | F (1, 20) = 13,34 | P=0,0016 |
|  | Food restriction | F (1, 20) = 43,60 | P<0,0001 |
| ***Sgk1*** | Interaction | F (1, 20) = 2,938 | P=0,1020 |
|  | Exercise | F (1, 20) = 0,2198 | P=0,6443 |
|  | Food restriction | F (1, 20) = 101,3 | P<0,0001 |
| ***Tnf-a*** | Interaction | F (1, 20) = 0,7497 | P=0,3968 |
|  | Exercise | F (1, 20) = 6,747 | P=0,0172 |
|  | Food restriction | F (1, 20) = 42,68 | P<0,0001 |
| ***Trem2*** | Interaction | F (1, 20) = 16,19 | P=0,0007 |
|  | Exercise | F (1, 20) = 18,89 | P=0,0003 |
|  | Food restriction | F (1, 20) = 18,89 | P=0,0003 |
| ***Iba1*** | Interaction | F (1, 20) = 4,247 | P=0,0526 |
|  | Exercise | F (1, 20) = 35,02 | P<0,0001 |
|  | Food restriction | F (1, 20) = 51,25 | P<0,0001 |
| ***Cd11b*** | Interaction | F (1, 20) = 1,272 | P=0,2728 |
|  | Exercise | F (1, 20) = 2,111 | P=0,1618 |
|  | Food restriction | F (1, 20) = 33,65 | P<0,0001 |
| ***Cd68*** | Interaction | F (1, 20) = 0,3326 | P=0,5706 |
|  | Exercise | F (1, 20) = 16,99 | P=0,0005 |
|  | Food restriction | F (1, 20) = 0,01141 | P=0,9160 |
| ***Cx3cr1*** | Interaction | F (1, 20) = 0,4677 | P=0,5019 |
|  | Exercise | F (1, 20) = 17,89 | P=0,0004 |
|  | Food restriction | F (1, 20) = 44,11 | P<0,0001 |
| **PND42** | **Two-way ANOVA** | ***F (DFn, DFd)*** | ***P value*** |
| **GR cytosol** | Interaction | F (1, 16) = 4,921 | P=0,0414 |
|  | Exercise | F (1, 16) = 3,955 | P=0,0641 |
|  | Food restriction | F (1, 16) = 14,35 | P=0,0016 |
| **GR nucleus** | Interaction | F (1, 16) = 25,00 | P=0,0001 |
|  | Exercise | F (1, 16) = 7,740 | P=0,0133 |
|  | Food restriction | F (1, 16) = 11,24 | P=0,0040 |
| **FKBP5** | Interaction | F (1, 16) = 15,41 | P=0,0012 |
|  | Exercise | F (1, 16) = 16,40 | P=0,0009 |
|  | Food restriction | F (1, 16) = 11,86 | P=0,0033 |
| ***Grα*** | Interaction | F (1, 16) = 0,5655 | P=0,4630 |
|  | Exercise | F (1, 16) = 11,70 | P=0,0035 |
|  | Food restriction | F (1, 16) = 20,52 | P=0,0003 |
| ***Grβ*** | Interaction | F (1, 16) = 7,029 | P=0,0174 |
|  | Exercise | F (1, 16) = 4,513 | P=0,0496 |
|  | Food restriction | F (1, 16) = 35,64 | P<0,0001 |
| ***Grα/ Grβ*** | Interaction | F (1, 16) = 8,976 | P=0,0085 |
|  | Exercise | F (1, 16) = 20,74 | P=0,0003 |
|  | Food restriction | F (1, 16) = 56,71 | P<0,0001 |
| ***Gilz*** | Interaction | F (1, 16) = 124,0 | P<0,0001 |
|  | Exercise | F (1, 16) = 12,12 | P=0,0031 |
|  | Food restriction | F (1, 16) = 59,98 | P<0,0001 |
| ***Fkbp5*** | Interaction | F (1, 16) = 4,730 | P=0,0450 |
|  | Exercise | F (1, 16) = 55,97 | P<0,0001 |
|  | Food restriction | F (1, 16) = 66,85 | P<0,0001 |
| ***Sgk1*** | Interaction | F (1, 16) = 2,093 | P=0,1672 |
|  | Exercise | F (1, 16) = 3,276 | P=0,0891 |
|  | Food restriction | F (1, 16) = 152,6 | P<0,0001 |
| ***Tnf-a*** | Interaction | F (1, 15) = 45,91 | P<0,0001 |
|  | Exercise | F (1, 15) = 65,16 | P<0,0001 |
|  | Food restriction | F (1, 15) = 8,277 | P=0,0115 |
| ***Trem2*** | Interaction | F (1, 16) = 41,61 | P<0,0001 |
|  | Exercise | F (1, 16) = 9,332 | P=0,0076 |
|  | Food restriction | F (1, 16) = 3,845 | P=0,0675 |
| ***Iba1*** | Interaction | F (1, 16) = 16,51 | P<0,001 |
|  | Exercise | F (1, 16) = 44,20 | P<0,001 |
|  | Food restriction | F (1, 16) = 83,12 | P<0,001 |
| ***Cd11b*** | Interaction | F (1, 16) = 4,595 | P=0,0478 |
|  | Exercise | F (1, 16) = 2,821 | P=0,1125 |
|  | Food restriction | F (1, 16) = 91,16 | P<0,0001 |
| ***Cd68*** | Interaction | F (1, 16) = 15,09 | P=0,0013 |
|  | Exercise | F (1, 16) = 1,932 | P=0,1835 |
|  | Food restriction | F (1, 16) = 5,643 | P=0,0304 |
| ***Cx3cr1*** | Interaction | F (1, 16) = 13,12 | P=0,0023 |
|  | Exercise | F (1, 16) = 8,521 | P=0,0100 |
|  | Food restriction | F (1, 16) = 49,96 | P<0,0001 |
| **PND49** | **Two-way ANOVA** | ***F (DFn, DFd)*** | ***P value*** |
| **GR cytosol** | Interaction | F (1, 16) = 0,02864 | P=0,8677 |
|  | Exercise | F (1, 16) = 2,668 | P=0,1219 |
|  | Food restriction | F (1, 16) = 0,1526 | P=0,7012 |
| **GR nucleus** | Interaction | F (1, 16) = 0,03685 | P=0,8502 |
|  | Exercise | F (1, 16) = 0,2569 | P=0,6191 |
|  | Food restriction | F (1, 16) = 1,763 | P=0,2029 |
| **FKBP5** | Interaction | F (1, 16) = 0,5615 | P=0,4646 |
|  | Exercise | F (1, 16) = 0,1574 | P=0,6968 |
|  | Food restriction | F (1, 16) = 2,005 | P=0,1760 |
| ***Grα*** | Interaction | F (1, 16) = 0,2262 | P=0,6408 |
|  | Exercise | F (1, 16) = 0,2487 | P=0,6248 |
|  | Food restriction | F (1, 16) = 0,4762 | P=0,5001 |
| ***Grβ*** | Interaction | F (1, 16) = 3,263 | P=0,0897 |
|  | Exercise | F (1, 16) = 0,07332 | P=0,7900 |
|  | Food restriction | F (1, 16) = 2,010 | P=0,1755 |
| ***Grα/ Grβ*** | Interaction | F (1, 16) = 4,624 | P=0,0472 |
|  | Exercise | F (1, 16) = 1,899 | P=0,1872 |
|  | Food restriction | F (1, 16) = 1,616 | P=0,2218 |
| ***Gilz*** | Interaction | F (1, 16) = 6,840 | P=0,0187 |
|  | Exercise | F (1, 16) = 77,13 | P<0,0001 |
|  | Food restriction | F (1, 16) = 27,90 | P<0,0001 |
| ***Fkbp5*** | Interaction | F (1, 16) = 4,555 | P=0,0486 |
|  | Exercise | F (1, 16) = 15,07 | P=0,0013 |
|  | Food restriction | F (1, 16) = 9,403 | P=0,0074 |
| ***Sgk1*** | Interaction | F (1, 16) = 5,334 | P=0,0346 |
|  | Exercise | F (1, 16) = 11,69 | P=0,0035 |
|  | Food restriction | F (1, 16) = 0,7016 | P=0,4146 |
| ***Tnf-a*** | Interaction | F (1, 16) = 10,92 | P=0,0045 |
|  | Exercise | F (1, 16) = 14,99 | P=0,0014 |
|  | Food restriction | F (1, 16) = 3,758 | P=0,0704 |
| ***Trem2*** | Interaction | F (1, 16) = 12,35 | P=0,0029 |
|  | Exercise | F (1, 16) = 9,599 | P=0,0069 |
|  | Food restriction | F (1, 16) = 0,005397 | P=0,9423 |
| ***Iba1*** | Interaction | F (1, 16) = 15,43 | P=0,0012 |
|  | Exercise | F (1, 16) = 5,590 | P=0,0310 |
|  | Food restriction | F (1, 16) = 12,38 | P=0,0029 |
| ***Cd11b*** | Interaction | F (1, 16) = 16,78 | P=0,0008 |
|  | Exercise | F (1, 16) = 24,85 | P=0,0001 |
|  | Food restriction | F (1, 16) = 22,58 | P=0,0002 |
| ***Cd68*** | Interaction | F (1, 16) = 8,214 | P=0,0112 |
|  | Exercise | F (1, 16) = 18,37 | P=0,0006 |
|  | Food restriction | F (1, 16) = 12,11 | P=0,0031 |
| ***Cx3cr1*** | Interaction | F (1, 16) = 0,3657 | P=0,5538 |
|  | Exercise | F (1, 16) = 40,01 | P<0,0001 |
|  | Food restriction | F (1, 16) = 7,240 | P=0,0161 |

**Supplementary table 13** Detailed statistical analysis of the Two-way ANOVA performed on data presented in Figure 4, panels a-f, related to protein levels and gene expression analysis.

|  |  | **Tuckey’s multiple comparison test** | |
| --- | --- | --- | --- |
| **PND40** |  | *Mean difference* | *Adjusted P value* |
| ***Grα*** | CTRL vs ABA | 0,1817 | 0,0003 |
|  | FR vs ABA | 0,1540 | 0,0019 |
|  | EXE vs ABA | 0,1660 | 0,0009 |
| ***Trem2*** | CTRL vs ABA | 0,004500 | <0,0001 |
|  | FR vs ABA | 0,004333 | <0,0001 |
|  | EXE vs ABA | 0,004333 | <0,0001 |
| **PND42** |  | *Mean difference* | *Adjusted P value* |
| **GR cytosol** | CTRL vs FR | 0,1627 | 0,0031 |
|  | CTRL vs EXE | 0,1140 | 0,0401 |
|  | CTRL vs ABA | 0,1565 | 0,0043 |
| **GR nucleus** | CTRL vs ABA | 0,1258 | 0,0026 |
|  | FR vs ABA | 0,1596 | 0,0003 |
|  | EXE vs ABA | 0,1713 | 0,0001 |
| **FKBP5** | CTRL vs ABA | -0,2465 | 0,0004 |
|  | FR vs ABA | -0,2623 | 0,0002 |
|  | EXE vs ABA | -0,2424 | 0,0004 |
| ***Grβ*** | CTRL vs FR | 0,02868 | 0,0088 |
|  | CTRL vs EXE | 0,02265 | 0,0421 |
|  | CTRL vs ABA | 0,04316 | 0,0002 |
| ***Grα/ Grβ*** | CTRL vs FR | 0,4177 | <0,0001 |
|  | CTRL vs EXE | 0,2996 | 0,0004 |
|  | CTRL vs ABA | 0,4795 | <0,0001 |
|  | EXE vs ABA | 0,1799 | 0,0255 |
| ***Gilz*** | CTRL vs FR | -0,5214 | <0,0001 |
|  | CTRL vs EXE | -0,2114 | 0,0003 |
|  | CTRL vs ABA | -0,1177 | 0,0372 |
|  | FR vs ABA | 0,4037 | <0,0001 |
| ***Fkbp5*** | CTRL vs FR | -0,3228 | 0,0031 |
|  | CTRL vs EXE | -0,2854 | 0,0085 |
|  | CTRL vs ABA | -0,8423 | <0,0001 |
|  | FR vs ABA | -0,5194 | <0,0001 |
|  | EXE vs ABA | -0,5568 | <0,0001 |
| ***Tnf-a*** | CTRL vs ABA | -0,7573 | <0,0001 |
|  | FR vs ABA | -1,027 | <0,0001 |
|  | EXE vs ABA | -0,6676 | <0,0001 |
| ***Trem2*** | CTRL vs FR | 0,2861 | 0,0001 |
|  | CTRL vs EXE | 0,3233 | <0,0001 |
|  | CTRL vs ABA | 0,1706 | 0,0129 |
|  | EXE vs ABA | -0,1527 | 0,0271 |
| ***Iba1*** | CTRL vs FR | 0,1534 | 0,01 |
|  | CTRL vs ABA | 0,4785 | <0,001 |
|  | CTRL vs ABA | 0,3251 | <0,001 |
|  | EXE vs ABA | 0,4001 | <0,001 |
| ***Cd11b*** | CTRL vs FR | 0,3812 | 0,0004 |
|  | CTRL vs ABA | 0,5780 | <0,0001 |
|  | EXE vs ABA | 0,6019 | <0,0001 |
| ***Cd68*** | CTRL vs FR | 0,2941 | 0,0022 |
|  | CTRL vs EXE | 0,2478 | 0,0089 |
| ***Cx3cr1*** | CTRL vs FR | 0,3025 | <0,0001 |
|  | CTRL vs EXE | 0,1851 | 0,0014 |
|  | CTRL vs ABA | 0,2826 | <0,0001 |
| **PND49** |  | *Mean difference* | *Adjusted P value* |
| ***Gilz*** | CTRL vs EXE | 0,1361 | 0,0025 |
|  | CTRL vs ABA | 0,3105 | <0,0001 |
|  | FR vs ABA | 0,2516 | <0,0001 |
|  | EXE vs ABA | 0,1743 | 0,0002 |
| ***Fkbp5*** | CTRL vs ABA | 0,1541 | 0,0008 |
|  | FR vs ABA | 0,1334 | 0,0031 |
|  | EXE vs ABA | 0,1154 | 0,0099 |
| ***Sgk1*** | CTRL vs ABA | 0,1493 | 0,0375 |
|  | FR vs ABA | 0,2009 | 0,0046 |
| ***Tnf-a*** | CTRL vs ABA | 0,3508 | 0,0041 |
|  | FR vs ABA | 0,4333 | 0,0006 |
|  | EXE vs ABA | 0,3166 | 0,0093 |
| ***Trem2*** | FR vs ABA | -0,6854 | 0,0013 |
| ***Iba1*** | CTRL vs ABA | -0,2997 | 0,0037 |
|  | FR vs ABA | -0,3206 | 0,0021 |
|  | EXE vs ABA | -0,3794 | 0,0004 |
| **Cd11b** | CTRL vs ABA | -1,342 | <0,0001 |
|  | FR vs ABA | -1,252 | <0,0001 |
|  | EXE vs ABA | -1,220 | <0,0001 |
| **Cd68** | CTRL vs ABA | -0,9197 | 0,0003 |
|  | FR vs ABA | -0,8469 | 0,0006 |
|  | EXE vs ABA | -0,7515 | 0,0019 |

**Supplementary table 14.** Detailed statistical values of the main effects of the Two-way ANOVA performed on behavioral measurements presented in Figure 5, panels b-d.

| **Two-way ANOVA** |  | ***F (DFn, DFd)*** | ***P value*** |
| --- | --- | --- | --- |
| **Distance increase** | Time | F (6, 108) = 3,348 | 0,0046 |
|  | Group | F (2,736, 49,24) = 62,57 | <0,0001 |
|  | Time x group | F (1, 18) = 0,1768 | 0,6791 |
| **Mean speed** | Time | F (3,050, 65,06) = 44,49 | <0,0001 |
|  | Group | F (1, 22) = 0,3260 | 0,5738 |
|  | Time x group | F (6, 128) = 3,971 | 0,0011 |
| **Long sequence count** | Time | F (6, 108) = 8,077 | P<0,0001 |
|  | Group | F (1,981, 35,66) = 57,63 | P<0,0001 |
|  | Time x group | F (1, 18) = 5,135 | P=0,0360 |

**Supplementary table 15.** Detailed statistical analysis of the Šidák’s multiple comparison test performed on behavioral measurements presented in Figure 3, panels b-d.

|  |  | **Šidák’s multiple comparison test** | |
| --- | --- | --- | --- |
| **Distance increase** |  | *Mean difference (g)* | *Adjusted P value* |
| **PND42** | ABA-SAL vs ABA-RU486 | 4727 | 0,0079 |
| **Mean speed** |  |  | |
| **PND42** | ABA-SAL vs ABA-RU486 | 5,406 | 0,0283 |
| **Sequence count** |  |  | |
| **PND42** | ABA-SAL vs ABA-RU486 | 460,1 | 0,0206 |

**Supplementary table 16.** Detailed statistical values of the main effects of the Two-way ANOVA related to corticosterone plasmatic levels showed in Figure 5 panel e and the One-way ANOVA conducted on data presented in Figure 5, panels e-i, k-n, related to blood and bone marrow analysis. Pearson’s product–moment correlation (r) analyses and linear regression analyses (R^2^) presented in Figure 5 panel f.

|  | **Two-way ANOVA** | ***F (DFn, DFd)*** | ***P value*** |
| --- | --- | --- | --- |
| **Corticosterone** | Interaction | F (1, 29) = 0,7488 | P=0,3940 |
|  | Group | F (1, 29) = 93,94 | P<0,0001 |
|  | Treatment | F (1, 29) = 36,23 | P<0,0001 |
| **One-way ANOVA** | **F (DFn, DFd)** | **p value** |  |
| **CD45 cell/ul blood** | F (2, 24) = 6,011 | P=0,0077 |  |
| **CD3 cell/ul blood** | F (2, 24) = 2,645 | P=0,0916 |  |
| **Bcells cell/ul blood** | F (2, 24) = 9,804 | P=0,0008 |  |
| **Granulocyte cell/ul blood** | F (2, 24) = 0,08329 | P=0,9204 |  |
| **CD45 cell/ul femur** | F (2, 25) = 0,8176 | P=0,4530 |  |
| **CD3 % of CD45 femur** | F (2, 25) = 43,45 | P<0,001 |  |
| **B cells % of CD45 femur** | F (2, 25) = 5,903 | P=0,0079 |  |
| **Granulocytes % of CD45 femur** | F (2, 25) = 6,084 | P=0,0070 |  |
| **Correlation** | **Pearson r** | **R^2^** | **p value** |
| **Distance travelled – Corticosterone**  **(ABA-SAL)** | 0,6703 | 0,4493 | 0,15 |
| **Distance travelled – Corticosterone**  **(ABA-RU486)** | -0,6831 | 0,4666 | 0,01 |

**Supplementary table 17.** Detailed statistical analysis of the Two-way ANOVA performed on data presented in Figure 5 panel e, and of the One-way ANOVA conducted on data presented in Figure 5, panels e-i, k-n, related to plasma, blood and bone marrow analysis.

| **Tuckey’s multiple comparison test** |  | **Mean differences** | **p value** |
| --- | --- | --- | --- |
| **CD45 cell/ul blood** | CTRL-SAL vs ABA-SAL | 701,3 | 0,0168 |
|  | CTRL-SAL vs ABA-RU486 | 672,6 | 0,0135 |
| **Bcells cell/ul blood** | CTRL-SAL vs ABA-SAL | 324,2 | 0,0023 |
|  | CTRL-SAL vs ABA-RU486 | 311,9 | 0,0017 |
| **CD3 % of CD45 femur** | CTRL-SAL vs ABA-SAL | -5,305 | <0,001 |
|  | CTRL-SAL vs ABA-RU486 | -3,422 | <0,001 |
|  | ABA-SAL vs ABA- RU486 | 1,883 | 0,004 |
| **Granulocytes % of CD45 femur** | CTRL-SAL vs ABA-SAL | -10,69 | 0,0323 |
|  | CTRL-SAL vs ABA-RU486 | -12,07 | 0,0073 |

**Supplementary table 18.** Detailed statistical values of the main effects of the One-way ANOVA conducted on data presented in Figure 5, panels o-q, s, t, related to protein levels and gene expression analysis.

| **One-way ANOVA** | **F (DFn, DFd)** | **p value** |
| --- | --- | --- |
| **GR cytosol** | F (2, 22) = 19,99 | P<0,001 |
| **GR nucleus** | F (2, 20) = 12,36 | P=0,0003 |
| **FKBP5** | F (2, 22) = 15,37 | P<0,0001 |
| ***Grα*** | F (2, 24) = 126,2 | P<0,0001 |
| ***Grβ*** | F (2, 23) = 23,63 | P<0,0001 |
| ***Grα/ Grβ*** | F (2, 23) = 9,585 | P=0,0009 |
| ***Gilz*** | F (2, 24) = 15,48 | P<0,0001 |
| ***Fkbp5*** | F (2, 24) = 81,97 | P<0,0001 |
| ***Sgk1*** | F (2, 24) = 206,0 | P<0,0001 |
| ***Tnf-a*** | F (2, 23) = 22,66 | P<0,0001 |
| ***Trem2*** | F (2, 24) = 25,31 | P<0,0001 |
| ***Iba1*** | F (2, 24) = 92,88 | P<0,0001 |
| ***Cd11b*** | F (2, 24) = 72,43 | P<0,0001 |
| ***Cd68*** | F (2, 24) = 22,82 | P<0,0001 |
| ***Cx3cr1*** | F (2, 23) = 5,234 | P=0,0134 |

**Supplementary table 19** Detailed statistical analysis of the One-way ANOVA and performed on data presented in Figure 5, panels o-q, s, t, related to protein levels and gene expression analysis.

|  |  | **Tuckey’s multiple comparison test** | |
| --- | --- | --- | --- |
|  |  | *Mean difference* | *Adjusted P value* |
| **GR cytosol** | CTRL-SAL vs ABA-SAL | 0,3759 | <0,001 |
|  | CTRL-SAL vs ABA-RU486 | 0,1861 | 0,004 |
|  | ABA-SAL vs ABA- RU486 | -0,1898 | 0,005 |
| **GR nucleus** | CTRL-SAL vs ABA-SAL | 0,3699 | 0,0007 |
|  | ABA-SAL vs ABA- RU486 | -0,3292 | 0,0006 |
| **FKBP5** | CTRL-SAL vs ABA-SAL | -0,5276 | 0,0006 |
|  | CTRL-SAL vs ABA-RU486 | -0,5321 | <0,0001 |
| ***Grα*** | CTRL-SAL vs ABA-SAL | 0,2823 | <0,0001 |
|  | CTRL-SAL vs ABA-RU486 | 0,1771 | <0,0001 |
|  | ABA-SAL vs ABA- RU486 | -0,1052 | <0,0001 |
| ***Grβ*** | CTRL-SAL vs ABA-SAL | 0,2098 | 0,0008 |
|  | CTRL-SAL vs ABA-RU486 | 0,3002 | <0,0001 |
| ***Grα/ Grβ*** | CTRL-SAL vs ABA-RU486 | -0,1433 | 0,0083 |
|  | ABA-SAL vs ABA- RU486 | -0,1685 | 0,0021 |
| ***Gilz*** | CTRL-SAL vs ABA-SAL | -0,3816 | <0,0001 |
|  | ABA-SAL vs ABA- RU486 | 0,2899 | 0,0005 |
| ***Fkbp5*** | CTRL-SAL vs ABA-SAL | -0,4029 | <0,0001 |
|  | CTRL-SAL vs ABA-RU486 | -0,5010 | <0,0001 |
| ***Sgk1*** | CTRL-SAL vs ABA-SAL | -0,5069 | <0,0001 |
|  | CTRL-SAL vs ABA-RU486 | -0,3732 | <0,0001 |
|  | ABA-SAL vs ABA- RU486 | 0,1337 | <0,0001 |
| ***Tnf-a*** | CTRL-SAL vs ABA-SAL | -0,2059 | 0,0195 |
|  | CTRL-SAL vs ABA-RU486 | -0,3975 | <0,0001 |
|  | ABA-SAL vs ABA- RU486 | -0,1916 | 0,0188 |
| ***Trem2*** | CTRL-SAL vs ABA-SAL | 0,09802 | 0,0315 |
|  | CTRL-SAL vs ABA-RU486 | 0,2237 | <0,0001 |
|  | ABA-SAL vs ABA- RU486 | 0,1257 | 0,0025 |
| ***Iba1*** | CTRL-SAL vs ABA-SAL | 0,3403 | <0,0001 |
|  | CTRL-SAL vs ABA-RU486 | 0,2520 | <0,0001 |
|  | ABA-SAL vs ABA- RU486 | -0,08827 | 0,0038 |
| ***Cd11b*** | CTRL-SAL vs ABA-SAL | 0,4692 | <0,0001 |
|  | CTRL-SAL vs ABA-RU486 | 0,4548 | <0,0001 |
| ***Cd68*** | CTRL-SAL vs ABA-RU486 | -0,2999 | 0,0001 |
|  | ABA-SAL vs ABA- RU486 | -0,3742 | <0,0001 |
| ***Cx3cr1*** | CTRL-SAL vs ABA-SAL | 0,2559 | 0,0165 |
|  | ABA-SAL vs ABA- RU486 | -0,2194 | 0,0271 |

**Supplementary figure 2.** Cropped immunoblot related to the expression levels of glucocorticoid receptor (GR) and FKBP5 measured in the cytosolic (A) and nuclear (B) fraction of the ventral hippocampus in controls (1), food restricted (2), exercise (3) and activity-based anorexia (4) rats at post-natal day (PND)40.

**Supplementary figure 3.** Cropped immunoblot related to the expression levels of glucocorticoid receptor (GR) and FKBP5 measured in the cytosolic ((A) and nuclear (B) fraction of the ventral hippocampus in controls (1), food restricted (2), exercise (3) and activity-based anorexia (4) rats at PND42.

**Supplementary figure 4.** Cropped immunoblot related to the expression levels of glucocorticoid receptor (GR) and FKBP5 measured in the cytosolic ((A) and nuclear (B) fraction of the ventral hippocampus in controls (1), food restricted (2), exercise (3) and activity-based anorexia (4) rats at PND42.

**Supplementary figure 5.** Cropped immunoblot related to the expression levels of glucocorticoid receptor (GR) and FKBP5 measured in the cytosolic (A) and nuclear (B) fraction of the ventral hippocampus in controls-saline (1), activity-based anorexia (ABA)-saline (2), and ABA treated with RU486 (3) and rats at post-natal day 42.

**Supplementary figure 6.** Gating strategy for blood analysis.

**Supplementary figure 7.** Gating strategy for bone marrow analysis.

**Supplementary table 20** Multiple linear regression analysis for BMI, body weight. The dependent variable was BMI, and the predictors included Group (AN T0 vs HC) and Age as a covariate. Analysis of Variance (ANOVA) shows the overall significance of the model and individual contributions of each predictor. Parameter estimates include the regression coefficients (β), 95% confidence intervals, t-values, and p-values. Goodness of fit is indicated by R², reflecting the proportion of variance explained by the model. Multicollinearity was assessed using the variance inflation factor (VIF), indicating no problematic correlation between predictors. Age did not emerge as a significant confounding factor.

| **Model – Multiple linear regression analysis** | | | **Dependent variable: BMI** |  |  |  |
| --- | --- | --- | --- | --- | --- | --- |
| Analysis of Variance | SS | DF | MS | F (DFn, DFd) | P value |  |
| Regression | 397,3 | 2 | 198,6 | F (2, 62) = 39,14 | P<0,0001 |  |
| Group | 289,8 | 1 | 289,8 | F (1, 62) = 57,11 | P<0,0001 |  |
| Age | 13,12 | 1 | 13,12 | F (1, 62) = 2,585 | P=0,1130 |  |
| Residual | 314,6 | 62 | 5,075 |  |  |  |
| Total | 711,9 | 64 |  |  |  |  |
|  |  |  |  |  |  |  |
| Parameter estimates | Variable | Estimate | 95% CI (profile likelihood) | \|t\| | P value | P value summary |
| β0 | Intercept | 24,77 | 20,32 to 29,22 | 11,12 | <0,0001 | **** |
| β1 | Group[AN T0] | -5,783 | -7,312 to -4,253 | 7,557 | <0,0001 | **** |
| β2 | Age | -0,1379 | -0,3093 to 0,03355 | 1,608 | 0,1130 | ns |
|  |  |  |  |  |  |  |
| Goodness of Fit |  |  |  |  |  |  |
| Degrees of Freedom | 62 |  |  |  |  |  |
| R squared | 0,5580 |  |  |  |  |  |
|  |  |  |  |  |  |  |
| Multicollinearity | Variable | VIF | R2 with other variables |  |  |  |
| β0 | Intercept |  |  |  |  |  |
| β1 | Group[AN T0] | 1,800 | 0,4444 |  |  |  |
| β2 | Age | 1,800 | 0,4444 |  |  |  |
|  |  |  |  |  |  |  |
| **Model – Multiple linear regression analysis** | | | **Dependent variable:  body weight** |  |  |  |
| Analysis of Variance | SS | DF | MS | F (DFn, DFd) | P value |  |
| Regression | 2656 | 2 | 1328 | F (2, 62) = 30,31 | P<0,0001 |  |
| Group | 1932 | 1 | 1932 | F (1, 62) = 44,10 | P<0,0001 |  |
| Age | 85,57 | 1 | 85,57 | F (1, 62) = 1,953 | P=0,1672 |  |
| Residual | 2716 | 62 | 43,81 |  |  |  |
| Total | 5372 | 64 |  |  |  |  |
|  |  |  |  |  |  |  |
| Parameter estimates | Variable | Estimate | 95% CI (profile likelihood) | \|t\| | P value | P value summary |
| β0 | Intercept | 66,87 | 53,79 to 79,96 | 10,22 | <0,0001 | **** |
| β1 | Group[AN T0] | -14,93 | -19,42 to -10,44 | 6,641 | <0,0001 | **** |
| β2 | Age | -0,3521 | -0,8557 to 0,1515 | 1,398 | 0,1672 | ns |
|  |  |  |  |  |  |  |
| Goodness of Fit |  |  |  |  |  |  |
| Degrees of Freedom | 62 |  |  |  |  |  |
| R squared | 0,4944 |  |  |  |  |  |
|  |  |  |  |  |  |  |
| Multicollinearity | Variable | VIF | R2 with other variables |  |  |  |
| β0 | Intercept |  |  |  |  |  |
| β1 | Group[AN T0] | 1,800 | 0,4444 |  |  |  |
| β2 | Age | 1,800 | 0,4444 |  |  |  |
|  |  |  |  |  |  |  |
| **Model – Multiple linear regression analysis** | | | **Dependent variable:**  **EED-19 score** |  |  |  |
| Analysis of Variance | SS | DF | MS | F (DFn, DFd) | P value |  |
| Regression | 9,368 | 2 | 4,684 | F (2, 52) = 10,02 | P=0,0002 |  |
| Group | 2,936 | 1 | 2,936 | F (1, 52) = 6,281 | P=0,0154 |  |
| Age | 0,6981 | 1 | 0,6981 | F (1, 52) = 1,493 | P=0,2272 |  |
| Residual | 24,31 | 52 | 0,4674 |  |  |  |
| Total | 33,68 | 54 |  |  |  |  |
|  |  |  |  |  |  |  |
| Parameter estimates | Variable | Estimate | 95% CI (profile likelihood) | \|t\| | P value | P value summary |
| β0 | Intercept | 2,134 | 0,7067 to 3,561 | 3,000 | 0,0041 | ** |
| β1 | Group[AN T0] | 0,6092 | 0,1214 to 1,097 | 2,506 | 0,0154 | * |
| β2 | Age | -0,03337 | -0,08817 to 0,02143 | 1,222 | 0,2272 | ns |
|  |  |  |  |  |  |  |
| Goodness of Fit |  |  |  |  |  |  |
| Degrees of Freedom | 52 |  |  |  |  |  |
| R squared | 0,2782 |  |  |  |  |  |
|  |  |  |  |  |  |  |
| Multicollinearity | Variable | VIF | R2 with other variables |  |  |  |
| β0 | Intercept |  |  |  |  |  |
| β1 | Group[AN T0] | 1,710 | 0,4152 |  |  |  |
| β2 | Age | 1,710 | 0,4152 |  |  |  |
|  |  |  |  |  |  |  |

**Supplementary table 21.** Baseline characteristics of the subpopulation (n=7) used for analyses presented in Figures 1n-v) relative to the total cohort (n=35). were compared.

Mann-Whitney U tests were performed for non-normally distributed continuous variables (Age, Body weight, BMI, and Cortisol). Unpaired Student’s t-test (two-tailed) was performed for the EED-19 score.

| **Mann-Whitney test**  **ANt0 (n=35) vs Ant0 (n=7)** | **Median differences** | **Mann-Whitney U** | **p value** |
| --- | --- | --- | --- |
| **Age** | -1,000 | 90 | 0,4848 |
| **Body weight** | -0,8500 | 132,5 | 0,9939 |
| **BMI** | -1,040 | 126 | 0,7590 |
| **Cortisol (ng/mL)** | -23,96 | 80 | 0,3705 |
| **Unpaired Student’s t test**  **(two tailed)**  **ANt0 (n=35) vs Ant0 (n=7)** | **Mean difference ± SEM** | **t, dF** | **p value** |
| **EED-19** | 0,3427 ± 0,4303 | t=0,7965, df=33 | 0,4315 |
